# Locus of Adhesion and Autoaggregation (LAA) pathogenicity island genes *hes* and *sisA* are involved in virulence and biofilm formation in LEE-negative Shiga toxin-producing *Escherichia coli* (STEC)

**DOI:** 10.1128/spectrum.03352-25

**Published:** 2026-06-15

**Authors:** Giovanni Milani, Claudia Cortimiglia, Gabriele Bellotti, Franco Lucchini, Pier Sandro Cocconcelli

**Affiliations:** 1 Department for Sustainable Food Process (DiSTAS), Università Cattolica del Sacro Cuore, Piacenza/Cremona, Italy; Agriculture and Agri-Food Canada, Alberta, Canada; Universidad de Buenos Aires, Buenos Aires, Argentina

**Keywords:** STEC, LAA PAI, virulence, adhesion, biofilm, *hes*, *sisA*

## Abstract

Shiga toxin-producing *Escherichia coli* (STEC) is a major foodborne pathogen capable of causing severe diseases such as hemolytic uremic syndrome (HUS), particularly in children. While the locus of enterocyte effacement (LEE) has long been associated with STEC virulence, an increasing number of LEE-negative strains are being linked to human infections. Some strains harbor the locus of adhesion and autoaggregation (LAA), a pathogenicity island comprising genes implicated in adhesion and biofilm formation. In this study, we investigated the role of two LAA-associated genes, *hes* and *sisA*, in the virulence of the LEE-negative STEC strain UC4224 isolated from raw milk cheese. Using lambda Red recombineering, we generated mutant strains with deletions in *stx1*, *stx2*, and/or the *hes-sisA* region. *Galleria mellonella* larvae were employed as an *in vivo* infection model to evaluate pathogenicity. Mutants were also assessed for their ability to produce biofilm and adhere to epithelial cell lines. Our results showed that deletion of *hes-sisA* significantly decreased larval mortality, biofilm formation, and adhesion to Caco-2 intestinal cells. These findings suggest that the *hes* and *sisA* genes may have a role in STEC colonization and persistence, representing potential determinants of human infection. This work highlights the importance of LAA-associated *hes* and *sisA* in the pathogenicity of LEE-negative STEC strains and suggests that alternative virulence factors beyond Shiga toxins are critical to disease progression. Further studies are warranted to explore the functional roles of these and other genes within the LAA region across different STEC backgrounds and to improve their contribution to human infection.

**IMPORTANCE:**

Shiga toxin-producing *Escherichia coli* (STEC) is the third most common cause of gastrointestinal illness in Europe. Cattle act as asymptomatic reservoirs, transmitting infection to humans mainly through contaminated raw milk and meat. Several outbreaks linked to raw milk products have been reported in recent years, prompting EU Rapid Alert System notifications, including in 2024. These infections represent a major public health and economic concern. In 2019, the European Food Safety Authority recommended enhanced surveillance and a broader assessment of STEC pathogenicity, emphasizing virulence factors beyond Shiga toxins and intimin. This study investigates a specific genomic region within a pathogenicity island to elucidate its role in virulence and biofilm formation. The findings highlight additional molecular markers that may contribute to STEC pathogenic potential. Incorporating these markers into molecular screening and surveillance can improve the identification of high-risk STEC clones, supporting more accurate risk assessment and targeted public health interventions.

## INTRODUCTION

Shiga toxin-producing *Escherichia coli* (STEC) is a significant cause of foodborne illness, leading to conditions ranging from mild gastroenteritis to hemorrhagic colitis and severe hemolytic uremic syndrome (HUS), especially in children below 5 years (1). This pathogen has been associated with several outbreaks mainly linked to the consumption of raw dairy and meat products, and vegetable-based foods. According to the last European Union One Health Zoonoses Report, the number of STEC found in food and isolated from humans increased by 130% in 2023 compared to 2020, rising from 4,446 to 10,217 cases, with a growth of outbreaks related to dairy products (2). This upward trend highlights STEC as an emerging public health concern and underscores the need to better understand its environmental persistence, survival in food, and virulence mechanisms (3).

Historically, STEC strains have been classified into serogroups, with the “top seven” (O157, O26, O45, O103, O111, O121, and O145) linked to higher pathogenicity. However, the genomic plasticity of *E. coli* makes serotyping alone an unreliable predictor of clinical outcomes (4). STEC pathogenicity is known to be primarily associated with Shiga toxins (5), classified into two types, namely Stx1 and Stx2, of which different subtypes have been described (Stx1a-Stx1e and Stx2a-Stx2l) (6). Although the most severe clinical cases have been mainly associated with some of these Stx variants (6), the outcome of the infection depends on other virulence factors and host-related factors such as the health status and age of the human host (7, 8). Some highly pathogenic STEC strains harbor the locus of enterocyte locus of enterocyte effacement (LEE), a pathogenicity island made by different key components including which plays a crucial role in enabling adherence to and damaging host intestinal epithelial cells (9, 10). The compromising of intestinal epithelial cells is further aggravated by the presence of Shiga toxins, leading to major complications of symptoms (11).

Although LEE-positive STEC strains have been identified as the cause of the most severe forms of gastrointestinal disorders and HUS, in some cases, these diseases have been linked to LEE-negative strains (12). In particular, the European Food Safety Authority (EFSA) reported an increase in the number of *eae*-negative STEC strains isolated from animals, food, and humans in 2023 compared to 2022 (2).

In 2017, Montero et al. characterized a novel 80 kb-long pathogenicity island in LEE-negative STEC strains, designated as the locus of adhesion and autoaggregation (LAA) (13). A large-scale genomic analysis performed on LEE-negative STEC strains isolated from humans, animals, and food revealed that among pathogenicity islands (PAIs) in LEE-negative STEC strains, the LAA PAI was the most prevalent and most associated with *stx* gene variants (*stx*1a, *stx*2a, *stx*2c, and *stx*2d), mainly linked to the most severe outcomes of infection (14). Several genes, including *hes*, *iha*, *lesP*, *pagC*, *tpsA*, *tpsB*, and *ag43,* can be recognized as markers for the four modules that make up the LAA, which can exist as a whole or partial structure (13). Some of the genes harbored by this region were studied for their involvement in adhesion to epithelial cells and colonization in a mouse model, hypothesizing that they may make these strains particularly resistant in food and confer pathogenicity traits (14). In particular, the *hes* gene was suggested to be the marker of the LAA PAI, as it had been detected in all strains with a complete LAA island and in most LEE-negative strains (14). Recently, this gene was also demonstrated to be involved in the formation of biofilm in a STEC O91 strain (15). In the first LAA module, the gene *sisA*, located in close proximity to *hes*, has been identified and, based on sequence homology, has been hypothesized to play a role in immunomodulation. The *sisA* gene is the homolog of *Shigella flexneri shiA,* a gene encoded by the SHI-2 pathogenicity island, linked to the induction of inflammation (16, 17). In particular, s*hiA* was suggested to mediate the suppression of the innate response during *Shigella* infections, without reducing invasion and cytotoxicity in the mouse lung model (17). The *shiA* homolog in *Escherichia coli* strains has been subjected to very few studies (18, 19). The study by Lloyd and colleagues (19) revealed the presence of two s*hiA* homolog genes, *sisA* and *sisB*, with differing percentages of amino acid identity (83.3% and 96.8%, respectively). *SisA* was found to be involved in suppressing the host immune response but contributed to a lesser extent to reducing the inflammatory response. They also observed that *sisA* was typical of extraintestinal pathogenic *Escherichia coli* strains, whereas *sisB* was found more in the enteric strains.

Despite ongoing efforts, few and sometimes conflicting data on the pathogenicity of LAA are available. Expanding our understanding of the biological role of this genomic region is of paramount importance.

In our previous work (20), starting from an LAA-positive STEC strain isolated from raw milk cheese, we generated mutant strains lacking *stx1*, *stx2,* or both genes. Pathogenicity was evaluated using the *Galleria mellonella* infection model, which revealed that the inactivation of Shiga toxins did not completely abolish the virulent potential of the strains, suggesting the involvement of additional factors responsible for the strain pathogenicity. In particular, we hypothesized that the persistence of pathogenicity could be linked to genes located within the LAA PAI. Based on this, we selected a specific portion of the LAA region to investigate its role in virulence and environmental survival. The aims of this study were (i) to use a genome engineering strategy to delete a portion of the first LAA PAI module, creating mutants with different combinations of deletions in *stx1*, *stx*2, and/or the selected LAA segment; (ii) to assess the pathogenicity of these modified strains in *Galleria mellonella* larvae and compare their LD_50_ values to evaluate the contribution of the targeted LAA region to virulence; (iii) to evaluate the involvement of a region of LAA PAI in adhesion to Caco-2 cell lines and biofilm on food manufacturing surfaces.

## RESULTS

### Construction of *hes* and *sisA* null mutants

To investigate the role of the *hes* and *sisA* genes, located within the LAA region, in pathogenicity and biofilm development, STEC strain UC4224 and its derivatives UC4176 (UC4224Δ*stx1::kan*, Kan^R^), UC4177 (UC4224Δ*stx2::cat*, Cm^R^), and UC4178 (UC4224Δ*stx1::kan* Δ*stx2::cat*, Kan^R^ Cm^R^) were subjected to a 5,485 bp deletion. Using the lambda Red recombineering, *hes-sisA*-deleted strains were successfully generated: UC4315 (UC4224 Δ *hes-sisA ::Tet*, Tet^R^), UC4316 (UC4224Δ*stx1::kan*, Δ *hes-sisA ::Tet,* Kan^R^ Tet^R^), UC4317 (UC4224Δ*stx2::cat*, Δ *hes-sisA ::Tet,* Cm^R^ Tet^R^), and UC4308 (UC4224Δ*stx1::kan* Δ*stx2::cat*, Δ *hes-sisA ::Tet,* Kan^R^ Cm^R^ Tet^R^) (Table 1). The substitution of this region with the tetracycline resistance cassette (*TetA*) was confirmed by PCR analysis and Sanger sequencing.

**TABLE 1:** Relevant features of *E. coli* mutants genomes obtained in this study^*a*^^,^^*b*^

Strain	UC4224	UC4315	UC4316	UC4317	UC4308
GC%	50.86	50.86	50.87	50.86	50.87
Total length of chromosome (bp)	5,047,333	5,044,181	5,043,035	5,042,707	5,044,665
*stx1*	+	+	−	+	−
*stx2*	+	+	+	−	−
*hes-sisA*	+	−	−	−	−
AMR genes (position in the chromosome)	−	*Tet*(C) (1,840,815–1,842,005)	*aph(3')-IIa* (712,487–713,279) *tet*(C) (1,839,667–1,840,857)	*catA1* (contig 2: 12,184..12,843) *tet*(C) (contig 1: 2,994,798..2,995,988)	*aph(3')-IIa* (2,051,511–2,052,303) *catA1* 1 (2,770,273–2,770,932) *tet*(C) (923,887–925,077)

^*a*^ The AMR genes, introduced during the recombineering, and related positions, were indicated, along with the detection of virulence factors modified in this and in the previous study (20). The set of virulence genes for each strain is detailed in Table S1.

^*b*^ “+” indicates the presence of the gene, whereas “−” indicates its absence.

### Identification of genetic modification by genome characterization

To verify that genome editing was restricted to the intended loci, whole-genome sequencing using both short- and long-read technologies was performed for all mutant strains. Hybrid assembly generated complete genomes for UC4315 and UC4316, while UC4317 and UC4308 resulted in two contigs each (UC4317: contig 1 = 4,994,754 bp; contig 2 = 47,935 bp; UC4308: contig 1 = 4,860,360 bp; contig 2 = 184,305 bp). In all cases, the assemblies also included two plasmids with the same length as those identified in the parental strain UC4224 (20). The main features of the *de novo* assemblies are summarized in Table 1, including the parental strain previously characterized.

The virulome of each strain (Table S1) was investigated with VirulenceFinder to assess whether unintended modifications occurred in other virulence-associated genes. Comparison between the parental strain UC4224 and the mutant strains revealed an identical virulence gene repertoire, except for the targeted deletions. In particular, the *hra* gene, encoding the heat-resistant agglutinin and corresponding to the *hes* gene described within the LAA pathogenicity island by Montero et al. (13), was detected only in UC4224 and was absent in all mutant strains. Notably, *sisA* was not detected by VirulenceFinder, as it is not included in the tool database.

As expected, UC4224 did not harbor AMR genes, whereas all mutant strains carried the resistance markers introduced during recombineering. Specifically, *tet(C*) replaced the *hes–sisA* region, while *aph(3')-IIa* and *catA1* substituted the *stx1* gene and the B subunit of *stx2*, respectively, according to the configurations reported in Table 1. Each AMR gene was present as a single chromosomal insertion.

**Fig 1:**
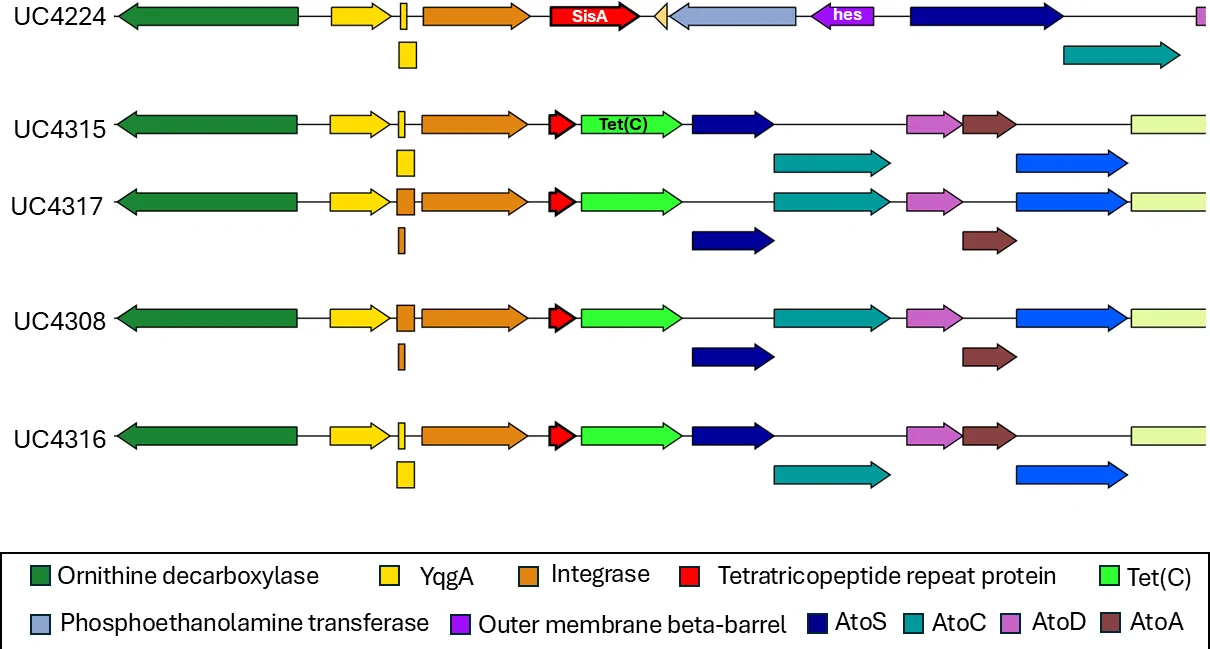
Genetic structure of the edited part of mutant strains in comparison with that of parental strain UC4224, obtained using the Comparative Systems service of BV-BRC v.3.57.26. Each protein is represented by arrow in different colors, with the original annotation of PGAP described in the lower part of the figure. Only the names of the sisA and hes proteins are present in the parental strain.

To assess whether additional genes were significantly affected by the genomic modification and to confirm the absence of unintended rearrangements, pairwise alignments between UC4224 and each mutant strain generated in this study were performed using MUMmer4. Structural variations identified, aside from those involving Shiga toxin regions, were located within a genomic segment corresponding to the LAA region. The 5,485 bp region, which includes tetratricopeptide repeat protein, phosphoethanolamine transferase, outer membrane beta-barrel protein, and signal transduction histidine-protein kinase AtoS, identified according to annotation based on the NCBI’s PGAP (Prokaryotic Genome Annotation Pipeline), was replaced with the tetracycline resistance cassette (Table S1 and Fig. 1). Homology analysis, guided by reference sequences reported by Montero et al., confirmed that the tetratricopeptide repeat protein corresponds to SisA, while the outer membrane beta-barrel protein corresponds to Hes. As shown in Fig. 1, the genetic makeup in the region of interest was conserved across all strains, except for the edited segment encompassing *sisA*, *hes*, and part of *atoS*. Specifically, the recombineering process resulted in the deletion of 744 bp of the 1,044 bp *sisA* gene, the complete removal of *hes*, and the deletion of 858 bp within *atoS*.

### The involvement of *hes* and *sisA* genes in the pathogenicity against *Galleria mellonella*

In this study, we evaluated the virulence of strains derived from an LAA-positive STEC strain in which *stx1, stx2,* and a portion of the LAA PAI were deleted. The mortality rate was assessed by injecting *G. mellonella* larvae with bacterial suspensions ranging from 10^1^ to 10^7^ CFU/10 µL of the four mutant strains, their respective LAA-positive counterparts, and *E. coli* BL21 as a negative control. Untreated larvae and those exposed to 0.1 M phosphate-buffered saline (PBS) and different doses of *E. coli* BL21 showed no significant mortality. Mortality rates observed in larvae treated with UC4224, UC4176 (Δ*stx1*), UC4177 (Δ*stx2*), and UC4178 (Δ*stx1*, Δ*stx2*) were consistent with the finding reported by Milani et al. (20).

**Fig 2:**
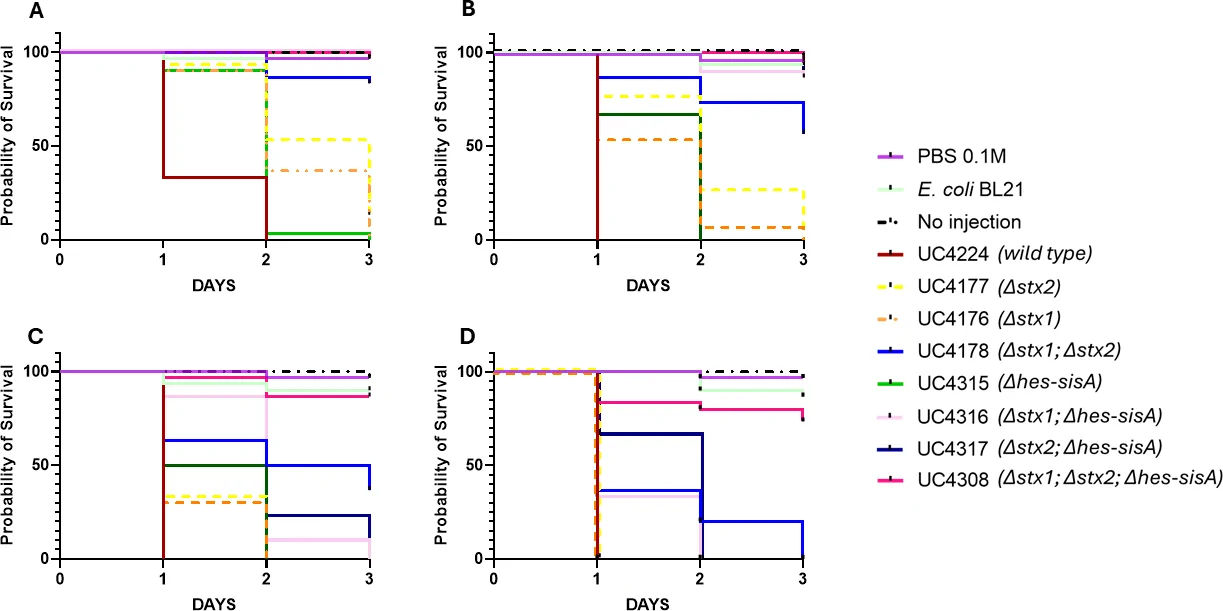
Kaplan-Meier survival curve of *Galleria mellonella* larvae injected with different loads of all strains, the parental strain UC4224 and mutant strains: 10^1^ CFU/10 µL (**A**), 10^2^ CFU/10 µL (**B**), 10^3^ CFU/10 µL (**C**), and 10^4^ CFU/10 µL (**D**). Larval survival was monitored for 24 h, 48 h, and 72 h. The results were obtained from triplicates of 10 larvae each. Larvae were also inoculated with negative controls, namely PBS and *E. coli* BL21.

The Kaplan-Meier survival analysis (Fig. 2) was performed on the four lowest doses (10^1^ to 10^4^ CFU/10 µL), since at concentrations above 4 log CFU/10 μL, the mortality rates for the UC4315 (Δ*hes-sisA*), UC4316 (Δ*stx1,* Δ*hes-sisA*), and UC4317 (Δ*stx2,* Δ*hes-sisA*) mutants were 100% after 24 h. When the two mutants UC4316 (Δ*stx1,* Δ*hes-sisA*) and UC4317 (Δ*stx2,* Δ*hes-sisA*) were tested, the obtained mortality rates were significantly lower than UC4315 (Δ*hes-sisA)* (*P* < 0.05) for the four lowest doses injected. UC4317 (Δ*stx2,* Δ*hes-sisA*) exhibited reduced pathogenicity compared to UC4316 (Δ*stx1,* Δ*hes-sisA*) (*P* < 0.05) exclusively at 10^4^ CFU/10 μL. Significant reductions in mortality rates were also observed when the LAA PAI null mutants were compared with their respective parental strain. In particular, UC4315 (Δ*hes-sisA*) was significantly less pathogenic than UC4224 (wild type) (*P* < 0.05) when injected with 10^1^ to 10^3^ CFU/10 µL. On the contrary, when *G. mellonella* larvae were injected with 10^4^ CFU/10 µL of UC4315 (Δ*hes-sisA*), the mortality rate was 100% after 24 h. Furthermore, when comparing the pathogenicity of UC4316 (Δ*stx1,* Δ*hes-sisA*) and UC4317 (Δ*stx2,* Δ*hes-sisA*) with their respective parents UC4176 (Δ*stx1*) and UC4177 (Δ*stx2*), a significant reduction in the mortality rate of *G. mellonella* was observed when treated with concentrations from 10^1^ to 10^4^ CFU/10 µL. Finally, UC4308 (Δ*stx1,* Δ*stx2, hes-sisA*) was revealed to be significantly (*P* < 0.05) less pathogenic than its originating strain UC4178 (Δ*stx1,* Δ*stx2*), when injected with loads of 10^2^ CFU/10 μL to 10^6^ CFU/10 μL. When treated with concentrations higher than 10^6^ CFU/10 μL, UC4308 (Δ*stx1,* Δ*stx2, hes-sisA*) showed 100% mortality after 24 h. Additionally, UC4308 (Δ*stx1,* Δ*stx2, hes-sisA*) was demonstrated to be significantly lower pathogenic than UC4315 (Δ*hes-sisA*), when inoculated at 10^1^ to 10^5^ CFU/10 µL. Moreover, we observed that UC4308 (Δ*stx1,* Δ*stx2, hes-sisA*) was significantly less pathogenic than UC4316 (Δ*stx1,* Δ*hes-sisA*) and UC4317 (Δ*stx2,* Δ*hes-sisA*) when the injected doses were 10^3^–10^4^ CFU/10 µL. Interestingly, at concentrations of 10^3^ CFU/10 μL or lower, no significant differences in mortality were observed between the UC4308 mutant and the non-pathogenic *E. coli* BL21 (*P* < 0.05).

**Fig 3:**
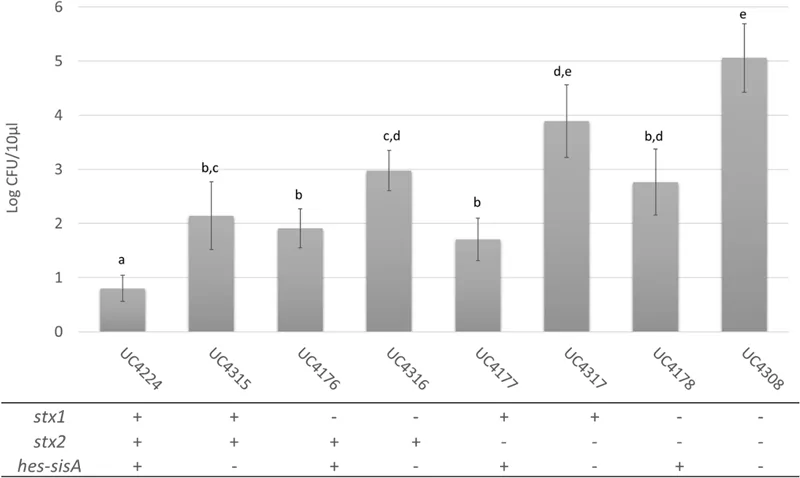
LD_50_ values calculated for the wild-type STEC UC4224 and its derivative mutants in the pathogenicity assay in *Galleria mellonella* larvae. Bars represent the mean values, and error bars indicate the 95% confidence intervals. Different letters above the bars indicate statistically significant differences between strains (*P* < 0.05).

Then, the probit regression model was employed to determine the LD_50_ after 24 h for all tested strains. Overall, we observed an increase in the LD_50_ as additional virulence factors were deleted. Among the strains, UC4224 exhibited the lowest LD₅₀, consistent with previous findings (20) (Fig. 3). The UC4315 mutant (Δ*hes-sisA*), which still harbored both intact *stx* operons, showed an LD_50_ of 1.4 × 10^2^ CFU/10 μL, significantly higher than that of the wild type UC4224 (*P* < 0.05). No statistically significant differences were observed in the LD₅₀ values of the mutants lacking *stx1* (UC4176), *stx2* (UC4177), or the *hes-sisA* region (UC4315).

The deletion of a portion of the LAA PAI from UC4176 (Δ*stx1*) to generate UC4316 (Δ*stx1,* Δ*hes-sisA*) resulted in a significant increase in LD_50_, reaching 9.4 × 10^2^ CFU/10 μL. Furthermore, strain UC4317 (Δ*stx2,* Δ*hes-sisA*) exhibited an LD_50_ of 7.7 × 10^3^ CFU/10 μL, representing two logarithmic orders increase compared to UC4177 (Δ*stx2*), highlighting a statistically significant difference (*P* < 0.05) in the number of cells causing the death of larvae. Notably, the LD_50_ of UC4317 (Δ*stx2,* Δ*hes-sisA*) was also significantly higher than that of UC4178 (Δ*stx1*, Δ*stx2*), which lacks both *stx* genes, suggesting a cumulative impact of the additional LAA deletion. No statistically significant difference in LD_50_ was observed between UC4316 (Δ*stx1,* Δ*hes-sisA*) and UC4317 (Δ*stx2,* Δ*hes-sisA*).

The concomitant absence of both *stx* and *hes-sisA* genes in UC4308 (Δ*stx1,* Δ*stx2,* Δ*hes-sisA*) resulted in a further increase in LD_50_ to 1.4 × 10^5^ CFU/10 μL, making it significantly less pathogenic than all the other strains, excluding UC4317 (Δ*stx2,* Δ*hes-sisA*). Although the difference between LD_50_ values of UC4308 (Δ*stx1,* Δ*stx2,* Δ*hes-sisA*) and UC4317 (Δ*stx2,* Δ*hes-sisA*) was not significant, it is noteworthy that the pathogenicity of UC4308 (Δ*stx1,* Δ*stx2,* Δ*hes-sisA*) was reduced by more than one Log CFU/10 μL.

These *in vivo* results indicated higher survival rates for larvae treated with mutants in the 5 kb region of LAA PAI than those exposed to their respective strains harboring *sisA-hes* genes. This is particularly evident for UC4308, where deletions of the *stx1*, *stx2*, *hes*, and *sisA* genes resulted in a marked reduction in pathogenicity.

### Involvement of *hes* and *sisA* genes in biofilm production

**Fig 4:**
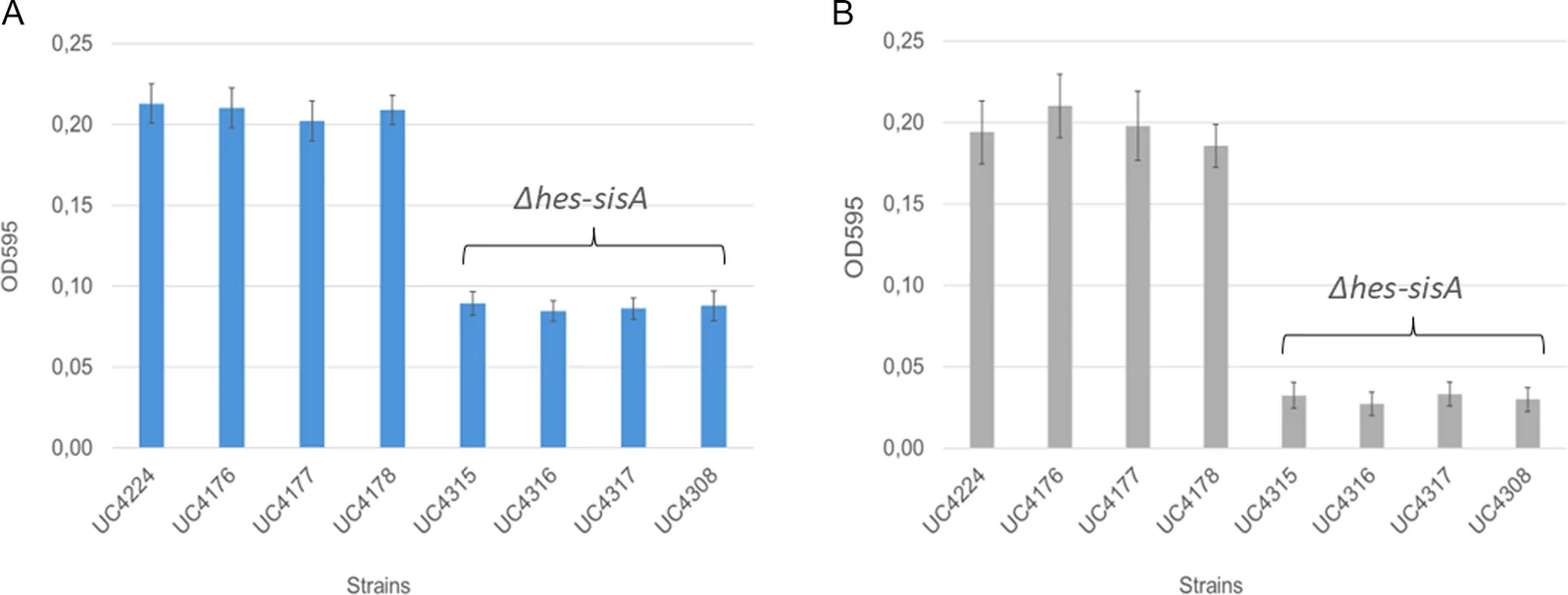
Biofilm formation of the wild type UC4224 and related mutants. The blue series (**A**) is related to the formation of biofilm on plastics, while the grey series (**B**) is related to the biofilm production on stainless steel. The quantification of biofilm was assessed by OD595. Error bars indicate standard deviation.

The ability of the wild-type STEC UC4224 strain and the six related mutants to produce biofilm on polystyrene and at the milk/stainless steel (SS) interface, representing the typical conditions of dairy production, was assessed. No significant differences (*P* < 0.05) in biofilm production on both types of surfaces were observed for UC4224 and the single and double mutants in Shiga toxin genes. In contrast, UC4315 (Δ*hes-sisA*), UC4316 (Δ*stx1,* Δ*hes-sisA*), UC4317 (Δ*stx2,* Δ*hes-sisA*), and UC4308 (Δ*stx1,* Δ*stx2,* Δ*hes-sisA*) strains, lacking the *hes* and *sisA* genes, exhibited a complete loss of this ability (Fig. 4). The biofilm production levels in these mutants were comparable to those of the negative control and significantly lower than those of strains retaining an intact LAA PAI region.

### Contribution of *hes* and *sisA* on Caco-2 cell adhesion

**Fig 5:**
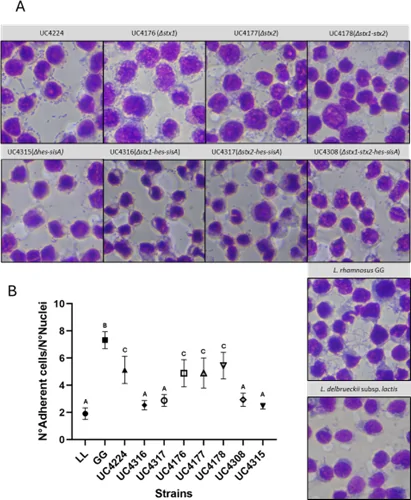
Adhesion of *Escherichia coli* UC4224 and related mutants to Caco-2 cells. (**A**) Representative images illustrate the adhesion of the wild-type strain UC4224 and its mutants carrying deletions in *stx1*, *stx2*, *hes-sisA*, or their combinations. Reference strains *L. rhamnosus* GG and *L. delbrueckii* subsp. *lactis* were included. (**B**) The graph reports the mean ± standard deviation of the number of adherent bacteria per nucleus for each strain. For each strain, the assay was performed in triplicate, considering 15 randomly selected. *L. delbrueckii* subsp. *lactis* (DSM 20072) and *L. rhamnosus* GG (ATCC 53103) were used as positive and negative controls, respectively. Error bars indicate standard deviation between triplicates. Letters represent significant differences between means (*P* < 0.05).

The role of the *hes* and *sisA* genes in Caco-2 cell adhesion was assessed for the STEC strain UC4224 and its seven mutants (Fig. 5A). The highest adhesion levels were observed for *Lacticaseibacillus rhamnosus* GG (ATCC 53103), with statistically significant differences observed (*P* < 0.05). A significant reduction in adhesion ability (*P* < 0.05) was noted in mutants lacking the *hes* and *sisA* genes (UC4315, UC4316, and UC4308) compared to strains UC4224, UC4176 (Δ*stx1*), UC4177 (Δ*stx2*), and UC4178 (Δ*stx1*, Δ*stx2*), which retain these genes (Fig. 5B). No significant differences were observed among strains with an intact LAA-PAI region. Similarly, no statistically significant differences in adhesion were found among UC4315 (Δ*hes-sisA*), UC4316 (Δ*stx1,* Δ*hes-sisA*), UC4317 (Δ*stx2,* Δ*hes-sisA*), and UC4308 (Δ*stx1,* Δ*stx2,* Δ*hes-sisA*) and *Lactobacillus delbrueckii* subsp. *lactis* (DSM 20072).

## DISCUSSION

LEE-negative STEC strains represent an important group of pathogenic bacteria that lack a genomic region typically associated with the virulence of enteropathogenic and enterohemorrhagic *E. coli* (21). Albeit the absence of the LEE locus, these strains are able to employ alternative virulence mechanisms to adhere to and invade host tissues. Recently, there has been increasing scientific interest in the investigation of LEE-negative STEC strains due to their association with severe diseases (22, 23). Indeed, the number of LEE-negative STEC strains isolated from patients with HUS in Japan (12) and in some parts of Europe has been increasing (23, 24).

Some of these strains lacked the LEE locus but were found to harbor the LAA pathogenicity island, which contains genes encoding adhesion factors that contribute to their pathogenicity (13). The LAA PAI region was named by Montero and colleagues, as it contains several novel and known virulence factors associated with bacterial adhesion and autoaggregation properties (13). These genes are crucial for both tight adhesion to host cells and the structured architecture of biofilms. Biofilm development represents an adaptive response that allows STEC strains to endure unfavorable conditions, thereby enhancing their survival in different environments, such as food, and increasing the risk of host infection (25). This capability varies across and within strains and is influenced by factors beyond just the microbial strain involved. While the LEE locus has been deeply characterized, the function of the 80 genes on the LAA pathogenicity island has yet to be elucidated. Indeed, understanding the genetic backgrounds and virulence profiles of these LEE-negative strains is critical for developing effective diagnostic and therapeutic strategies to mitigate the risks associated with infections (26). Here, for the first time, we investigated the role of two genes, *hes* and *sisA,* both located within the recently described LAA PAI, in the adhesion and virulence of an LAA-positive STEC strain isolated from a semi-hard raw milk cheese. Although these findings are based on a single strain and should therefore be interpreted with caution, they provide initial insights into a genomic region that remains poorly characterized and warrants further investigation.

Building upon previously constructed *stx*-deleted derivatives (20), we successfully generated additional mutant strains carrying targeted deletions within a 5 kb region of the LAA PAI, including the *hes* and *sisA* genes. To evaluate the potential occurrence of off-target effects resulting from unintended genomic rearrangements during genome editing, all mutant strains were subjected to whole-genome sequencing using a hybrid approach. Subsequent analyses focused on the characterization of the virulome and resistome, as well as comparative genomic analysis between each mutant and the parental strain, revealing the lack of large-scale rearrangements, with deletions in the expected positions. Comparative analysis between the mutant strains and the parental strain confirmed that *sisA* and *hes* were inactivated by the targeted deletion, which also encompassed a small portion of the *atoS* gene. In *E. coli*, AtoS, together with AtoC, regulates the a*toDAEB* operon, known to be involved in short-chain fatty acid catabolism and in mediating chemotactic responses to compounds such as acetoacetate and spermidine (27). Currently, this pathway has not been directly associated with bacterial adhesion or virulence traits. However, although *atoS* is not known to be directly involved in adhesion or virulence, a potential indirect contribution to the observed phenotype cannot be excluded.

These mutated strains were then used to evaluate the contribution of these genes to virulence. First, the pathogenicity assessment in *G. mellonella* showed that their deletion caused a significant reduction in mortality rate following inoculation with the double mutants UC4316 (Δ*stx1,* Δ*hes-sisA*) or UC4317 (Δ*stx2,* Δ*hes-sisA*) compared with UC4315 (Δ*hes-sisA*). This observation is consistent with the current knowledge about the link between pathogenicity and the presence of *stx* genes (28, 29). Additionally, UC4316 (Δ*stx1,* Δ*hes-sisA*) and UC4317 (Δ*stx2,* Δ*hes-sisA*) caused lower mortality rates than their respective parental strains UC4176 (Δ*stx1*) and UC4177 (Δ*stx2*), which retain the *hes* and *sisA* genes. These data were confirmed by the reduction in mortality rate of the triple mutant UC4308 respect to the UC4178 strain, lacking *stx* genes and harboring the entire LAA PAI. Moreover, UC4308 was revealed to be less virulent with respect to UC4316 (Δ*stx1,* Δ*hes-sisA*) and UC4317 (Δ*stx2,* Δ*hes-sisA*). Overall, these results suggest that this region within module I of the LAA PAI significantly contributed to the virulence of the UC4224 strain.

The hypothesis is further supported by the analysis of LD_50_ values. As shown in Fig. 2, the LD_50_ value increased significantly by one log unit for strains lacking *hes* and *sisA*, compared to the parental strains. A progressive increase in LD_50_ was observed as additional virulence-associated genes were deleted. Specifically, the LD_50_ of double mutants was one log higher than that of the single mutants, while the triple mutant exhibited an LD_50_ value one log higher than the double mutants. These findings suggest a cumulative effect of gene deletions on the attenuation of virulence, indicating that *hes* and *sisA* may contribute to the pathogenic potential of this STEC strain.

Previously, *hes* was investigated by Montero et al. (13) for its involvement in the agglutination of sheep erythrocytes, autoaggregation, and biofilm formation. The involvement of *hes* in biofilm formation was further confirmed by another study, in which deletion of the gene resulted in reduced biofilm production and alterations in its structure (15). The ability to mediate adherence and form biofilm can be considered a virulence factor because facilitates the colonization and resistance to host immune defenses, other than persistence in hostile environments. Indeed, the deletion of *hes* may have attenuated the colonization potential, further lowering the pathogenic process. In addition, we deleted *sisA*, which was suggested to be involved in the downregulation of inflammatory responses in the host, as previously described in *Shigella* species. By potentially downregulating inflammatory responses and influencing innate immune cell functions, bacterial pathogens can avoid immune detection and enhance their persistence within the host. The simultaneous lack of *hes* and *sisA* may have reduced the ability of strains UC4315 (Δ*hes-sisA*), UC4316 (Δ*stx1,* Δ*hes-sisA*), UC4317 (Δ*stx2,* Δ*hes-sisA*), and UC4308 (Δ*stx1,* Δ*stx2,* Δ*hes-sisA*) to adhere to host tissue and to evade immune surveillance in the *Galleria mellonella* model, contributing to the observed attenuation in pathogenicity. Further investigations employing targeted gene deletion approaches may provide valuable insights into the specific functional role of the *sisA* gene.

We further explored the effect of *hes* deletion on biofilm formation. Consistent with findings from other studies using different strains, we observed that deletion of a region including *hes* led to a marked reduction in biofilm formation on both stainless steel and polystyrene surfaces (Fig. 3). This finding is particularly relevant in the context of recent trends. The increasing number of STEC infections reported by EFSA, along with the growing prevalence of LEE-negative strains isolated from different sources, may reflect selective pressure acting on STEC populations. These pressures could be promoting genomic reorganization, enhancing the adaptability and environmental persistence of these pathogens (8, 30, 31). Pathogenic *E. coli* strains exhibiting hybrid virulence profiles are being reported with increasing frequency. This trend suggests that evolutionary pressures are actively driving the emergence of diverse combinations of virulence factors, primarily through horizontal gene transfer, resulting in the formation of distinct virulence-associated genomic islands (32, 33). Notably, these events have also involved LAA-positive strains, as previously reported by other studies (14).

Adhesion of bacterial strains to intestinal epithelial cells is a key factor in the colonization and persistence of STEC within the human gut (34, 35). Wild-type and mutant strains carrying *hes* and *sisA* genes exhibited significantly greater adhesion to Caco-2 cells compared to strains lacking these genes, supporting their role in mediating attachment (Fig. 4). In contrast, previous studies using HEp-2 cells reported more variable outcomes, potentially due to strain-specific expression patterns or cell-type differences (36). Such discrepancies could be justified by the fact that additional genes may contribute to adhesion, and that this trait is likely influenced by both physiological and environmental conditions. Indeed, the expression of adhesins appears to be a coordinated process that depends on the specific strain background as well as the surrounding environment (37).

While our findings provide new insights, some limitations need to be acknowledged to properly frame their interpretation. The possibility of polar effects on neighboring genes within the LAA region should be taken into account, although the absence of additional structural alterations suggests that major downstream disruptions are unlikely. The combination of precise genome editing and comprehensive genomic validation provides valid support for the association between the deleted loci and the observed phenotypes.

Whole-genome sequencing and comparative analyses confirmed that genome editing was restricted to the intended loci and did not introduce additional mutations or large-scale rearrangements. However, the deletion involved also a portion of the *atoS* gene. Although it is not known to be implicated in adhesion- or virulence-related processes, its potential indirect contribution cannot be completely excluded.

These results were obtained from a single LAA PAI-positive STEC strain, but additional data from different STEC strains carrying different combinations of LAA PAI modules are required to confirm these findings. Moreover, although the *G. mellonella* infection model provides a convenient and ethically acceptable system to assess bacterial virulence, some limitations should be acknowledged. Indeed, it lacks an adaptive immune system, and thus cannot fully reproduce the complex host-pathogen interactions occurring in mammals. Moreover, physiological differences, including gut environment, body temperature regulation, and tissue organization, may influence bacterial colonization and the expression of virulence factors. While larvae can be maintained at 37°C, prolonged exposure to this temperature may affect their immune competence and survival, potentially introducing variability. Finally, heterogeneity in larval age, size, and rearing conditions can affect reproducibility across experiments (38).

In conclusion, our preliminary study provides significant insights into the pathogenic potential of LEE-negative STEC strains, particularly those harboring the LAA pathogenicity island. The findings indicate that even in the absence of the conventional LEE locus, these strains can employ alternative virulence mechanisms, underscored by the critical roles of the *hes* and *sisA* genes, which enhance adherence and pathogenicity. The reduced virulence and increased LD_50_ values observed in the double and triple mutants highlight the cumulative impact of gene deletions on pathogenicity, emphasizing the importance of LAA-positive strains in the overall virulence profile.

Furthermore, our observations on adhesion to intestinal epithelial cells reinforce the essential role of these genes in promoting colonization and persistence of LEE-negative STEC in the gastrointestinal tract. In addition, we demonstrated a correlation between the absence of a specific segment encompassing the *hes* and *sisA* genes of the LAA pathogenicity island and reduced biofilm formation at the milk/stainless steel interface, a feature particularly relevant to the persistence of these strains in dairy processing environments.

This work expands our understanding of the genetic and functional diversity of pathogenic mechanisms employed by STEC strains and emphasizes the need for further research to uncover the breadth of virulence factors encoded by the LAA PAI. Such knowledge is crucial for developing effective diagnostic and therapeutic strategies to manage infections associated with these emerging pathogens. Given the increasing isolation of LEE-negative strains from severe disease cases, deepening our understanding of their virulence determinants is critical for public health and clinical interventions. However, further studies involving additional LAA-positive STEC strains with different combinations of virulence factors are needed to validate and extend these findings, as well as to assess their broader applicability across different genetic backgrounds. Moreover, the use of complementary infection models could provide valuable insights into the role of specific LAA-associated genes in host-pathogen interactions. In particular, evaluating these and additional *hes* and *sisA* mutant strains in human-derived intestinal enteroid infection systems may help to better recapitulate the intestinal environment and clarify the contribution of these genes to epithelial colonization, immune modulation, and overall pathogenicity (39).

## MATERIALS AND METHODS

### Bacterial strains, plasmids, and media

Bacterial strains used in this study are listed and described in Table 2. UC4224 STEC strain, originally isolated from semi-hard raw milk cheese of bovine origin and belonging to the O174:H2 serogroup, was grown in Luria-Bertani (LB) broth (Sigma-Aldrich). The mutated strains UC4176 (UC4224Δ*stx1*), UC4177 (UC4224Δ*stx2*), and UC4178 (UC4224Δ*stx1-*Δ*stx2*) were grown in LB broth supplemented with chloramphenicol (Cm) (Sigma-Aldrich) (50 μg/mL), kanamycin (Kan) (Sigma-Aldrich) (50 μg/mL), and tetracycline (Sigma-Aldrich) (3.5–10 μg/mL) when needed (20).

**TABLE 2:** Bacterial strains and plasmids used in this study^*a*^

Strain	Relevant genotype, phenotype	Reference/Source
*E. coli* UC4224	STEC parental strain	(20)
*E. coli* UC4315	UC4224 Δ*hes-sisA*::Tet, Tet^R^	This study
*E. coli* UC4176	UC4224Δ*stx1*::kan, Kan^R^	(20)
*E. coli* UC4316	UC4224Δ*stx1*::kan, Δ*hes-sisA*::Tet, Kan^R^ Tet^R^	This study
*E. coli* UC4177	UC4224Δ*stx2*::cat, Cm^R^	(20)
*E. coli* UC4317	UC4224Δ*stx2*::cat, Δ*hes-sisA*::Tet, Cm^R^ Tet^R^	This study
*E. coli* UC4178	UC4224Δ*stx1*::kan, Δ*stx2*::cat, Kan^R^ Cm^R^	(20)
*E. coli* UC4308	UC4224Δ*stx1*::kan, Δ*stx2*::cat, Δ*hes-sisA*::Tet, Kan^R^ Cm^R^ Tet^R^	This study
*E. coli* BL21	Negative control in pathogenicity assay	DSM 102053
*Lactobacillus delbrueckii* subsp. *lactis*	Negative control in Caco-2 adhesion assay	DSM 20072
*Lacticaseibacillus rhamnosus* – GG	Positive control in Caco-2 adhesion assay	ATCC 53103
Plasmids		
pSIM6	Plasmid expressing Lambda red recombination genes below the control of CI857 repressor, AmpR (Ts)	(40)
pACYC184	Template plasmid for the amplification of FRT-Tet-FRT amplicon, TetR CmR	(41)

^*a*^ Amp, ampicillin; Tet, tetracycline; Kan, kanamycin; Cm, chloramphenicol; superscripts “R” represent resistance.

The *E. coli* strain DH5α, used for the propagation and purification of plasmids, was grown in LB broth supplemented with ampicillin (Amp) (Sigma-Aldrich) (100 μg/mL). Lactobacillaceae were propagated in De Man-Rogosa-Sharpe medium (Oxoid) under anaerobic conditions at 37°C.

### Construction of the amplimer containing the tetracycline resistance cassette for the partial deletion of LAA PAI

A 5,485 bp region of the LAA PAI, encompassing the *hes* and *sisA* genes, was chosen as the target to be substituted by the tetracycline cassette. The strategy to obtain the fragment for the recombineering was previously described (20, 41, 42). Briefly, the tetracycline resistance cassette was first amplified from plasmid pACYC184 using primers TC-5 and TC-3. Then, primers LAA-UP/Tet-5′LAA and Tet-3′LAA/LAA-Down were designed for the amplification of a 373 bp upstream region (homologous arm UP) and 350 bp of LAA PAI downstream region (homologous arm DOWN), respectively. The tetracycline resistance cassette was then amplified with primers Tet-5′LAA and Tet-3′LAA, to obtain the cassette with upstream and downstream homologous regions. Ultimately, primers LAA-UP and LAA-DOWN were used to obtain the final fragment of 2,066 bp. The primers used are listed in Table 3.

**TABLE 3:** Oligonucleotides used in this study

Primer	Sequence (5′−3′)	Reference
Tc-5	TCAGCCCCATACGATATAAG	(42)
Tc-3	TGGAGTGGTGAATCCGTTAG	
LAA-UP	GCAACGTTAATCCCGGACAA	This study
Tet-5′LAA	CTTATATCGTATGGGGCTGACCAACTGCCAGCCCTTTAAG	
Tet-3′LAA	CTAACGGATTCACCACTCCACGCGGACACTTAACGATCTG	This study
LAA-DOWN	GGTTTCTTTGCGGGCAGTTA	

All amplified fragments were loaded on an electrophoresis gel and purified with NucleoSpin Gel and PCR Clean-up (Macherey-Nagel). After the purification of the final fragment, a second round of PCR was conducted using the same primer pair to increase the amount of amplicon.

For the primer design, Primer3web version 4.1.0 (https://primer3.ut.ee/) was used, and the specificity was checked against the fragment to be amplified and against the STEC UC4224 genome.

All the PCR reactions were performed using Phusion Flash High-Fidelity PCR Master Mix (Thermo Fisher Scientific) following the manufacturer’s instructions.

### Transformation with plasmids pSIM6, recombineering, and replacement confirmation

The strategy for the transformation and recombineering is described in detail in previous works (20).

Recombinants were identified after 1 to 2 days of incubation at 37°C. The resulting mutants were UC4315 (UC4224 Δ*hes-sisA::Tet*, Tet^R^), UC4316 (UC4224Δ*stx1::kan*, Δ*hes-sisA::Tet,* Kan^R^ Tet^R^), UC4317 (UC4224Δ*stx2::cat*, Δ*hes-sisA::Tet,* Cm^R^ Tet^R^), and UC4308 (UC4224Δ*stx1::kan* Δ*stx2::cat*, Δ*hes-sisA::Tet,* Kan^R^ Cm^R^ Tet^R^) (Table 2).

The obtained recombinants were cured as previously described (20). The correct substitution was confirmed by a locus-specific PCR using the primers LAA-UP and LAA-DOWN (Table 2), which provide amplicons differing in size between the parental strain and the respective recombinant. PCR products were sequenced by a commercial facility (Eurofins Genomics, Italy) using Sanger technology.

### Whole-genome sequencing and bioinformatic analyses

Mutant strains UC4315, UC4316, UC4317, and UC4308 were inoculated in LB broth with antibiotics as previously mentioned. Genomic DNA (gDNA) was extracted with E.Z.N.A. Bacterial DNA Kit (Omega Bio-tek), following the manufacturer’s instructions. Short-read sequencing was performed by Biodiversa srl (Treviso, Italy) using Illumina MiSeq platform with 150 paired-end run, while long reads were obtained with Oxford Nanopore Technology (ONT) using MinION Mk1B platform (Nanopore, Oxford, UK). For each strain, gDNA ONT libraries were prepared using the Rapid Barcoding Kit 24 v.14 (SQK-RBK114.24, ONT, UK) and loaded on the R10.4.1 flow cell (FLO-MIN114) and sequenced with MinKNOW v.23.11.5 software. FastQC v.0.12.1 software was used to evaluate the quality of raw reads. Hybrid assembly was performed with Unicycler v.0.5.1 (43) and Prokka v.1.14.6 (44) was used for annotation. Assemblies were deposited under the BioProject ID PRJNA1447475.

Genomes were further analyzed for virulence genes using VirulenceFinder v.2.0.5 of Center for Genomic Epidemiology (CGE) (https://cge.food.dtu.dk/services/VirulenceFinder/) to check the presence of *stx1* and *stx2* in comparison with the UC4224 parental strain and with ResFinder v.4.6.0 tool of CGE (https://genepi.food.dtu.dk/resfinder) to detect the presence of antimicrobial resistance genes inserted into mutant strains. Moreover, a comparison among the genomes of mutant strains and the parental strain was performed with MUMmer v4.0.1 (45) to identify gaps or SNPs.

### Pathogenicity assessment in *Galleria mellonella*

The pathogenicity of new mutants UC4315 (Δ*hes-sisA*), UC4316 (Δ*stx1,* Δ*hes-sisA*), UC4317 (Δ*stx2,* Δ*hes-sisA*), and UC4308 (Δ*stx1,* Δ*stx2,* Δ*hes-sisA*) was tested *in vivo* using *Galleria mellonella* larvae with the protocol previously described (20, 46). The strains UC4224 (wild type), UC4176 (Δ*stx1*), UC4177 (Δ*stx2*), and UC4178 (Δ*stx1,* Δ*stx2*) were also tested, other than BL21 and PBS 0.1 M as negative controls. Three independent biological replicates of 10 larvae were injected with 10 µL of 10-fold dilutions (from 10^1^ to 10^7^ CFU for each larva). The survival rate of each strain at each injection doses was determined using Kaplan-Meier survival curves provided by GraphPad Prism (Survival Curve 8.4.3). The significant differences in probability of survival among all strains were generated using log-rank tests (*P* < 0.05), while the LD_50_ values were calculated using probit analysis, according to the methodology of Finney (1971) with a 95% confidence limit (47).

### Biofilm assay

The biofilm-forming ability of wild-type STEC UC4224 and its mutants was initially evaluated using 96-well polystyrene microtiter plates (Sarstedt, Nümbrecht, Germany) in triplicate, following a previously described protocol (15) with minor modifications. Overnight LB cultures grown at 37°C were adjusted to an optical density (OD) at 620 nm of 0.5, corresponding to approximately 2 × 10^8^ CFU/mL. A 10 µL aliquot of each culture was inoculated into 190 µL of reconstituted skim milk (Oxoid) and incubated at 37°C for 24 h. Plates were then further incubated for 48 h at 37 °C, after which they were washed three times with sterile distilled water, fixed with 100% methanol, and stained with 1% crystal violet. Excess stain was removed by repeated washing with distilled water, and biofilm formation was quantified by measuring the OD595 using a Multiskan EX microplate reader (Thermo Electron Corporation, Waltham, MA, USA).

Subsequently, the adhesion capacity on SS spheres was evaluated following Bassi et al. (48), with minor changes (48). Ten sterile SS spheres (5 mm diameter) were placed in a 50 mL tube containing 10 mL of reconstituted skim milk (Oxoid), inoculated with 50 μL of an overnight culture (1 × 10^9^ CFU/mL) for each strain. After 24 h incubation at 37°C, SS spheres were removed from the milk, washed three times with distilled water to eliminate unattached cells from the biofilm and stained with 0.1% crystal violet solution for 15 min. Excess crystal violet solution was removed by washing twice with distilled water. Spheres were subsequently resuspended with 3 mL of ethanol/acetone solution (80/20, vol/vol) and vortexed for 3 min to detach biofilm cells. Therefore, the OD at 595 nm of the resulting solution was determined. Three independent replicates were carried out for each strain.

Statistical analysis was conducted using one-way ANOVA followed by Tukey’s HSD test to identify significant differences in biofilm formation across strains.

### Caco-2 cell line preparation and adhesion assay

Enterocyte-like Caco-2 cells p25 (ATCC HTB 37) were grown in Dulbecco’s modified Eagle’s minimal essential medium (DMEM) (VWR International), supplemented with 10% (vol/vol) heat-inactivated fetal bovine serum (Euroclone, Pero, Italy), 2 mM L-glutamine (Euroclone), and gentamicin (50 µg/mL) (Euroclone). Incubation of cell lines was carried out at 37°C in a 95% (vol/vol) atmosphere with 5% (vol/vol) CO_2_. After reaching confluence, the concentration of Caco-2 monolayer was evaluated by trypsinizing the cells for 10 min at 37°C and counting them in a hemocytometer. For the adhesion assay, Caco-2 cell lines were seeded on glass coverslips located in 24-well tissue culture plates (Sarstedt). An amount of 500 µL containing 5 × 10^4^ cells was transferred to each well and incubated at 37°C in a 95% (vol/vol) atmosphere with 5% (vol/vol) CO_2_ for 2 weeks. The culture medium was replaced every 48 h.

Adhesion experiments with STEC UC4224 and its seven mutant strains to epithelial Caco-2 cells were carried out as previously described by Tuomola et al. (49) with some modifications. Briefly, 12 h prior to the assay, the culture medium of the Caco-2 cells was replaced with fresh antibiotic-free DMEM. The 2-week-old monolayers were washed twice with PBS (pH 7.4). Overnight bacterial cultures of the wild-type strain and mutant strains were adjusted to an OD_600_ of 0.5 in PBS, corresponding to approximately 2 × 10^8^ CFU/mL. *Lacticaseibacillus rhamnosus* GG (ATCC 53103) and *Lactobacillus delbrueckii* subsp. *lactis* (DSM 20072) were included as positive and negative controls, respectively. A total of 300 µL of each bacterial suspension was added to the Caco-2 monolayers and incubated at 37°C for 1 h. The monolayers were washed four times with PBS, fixed with methanol at room temperature for 10 min, and then stained with a 1:20 dilution of Giemsa stain solution (Merck, Darmstadt, Germany) for 30 min. Slides were washed, air-dried, and examined under oil immersion microscopy at ×100 magnification. Each bacterial strain was tested in triplicate, and bacterial adhesion was quantified by counting adherent cells in 15 randomly selected microscopic fields per slide. Bacterial adhesion was normalized to the number of host cell nuclei per field. Statistical differences between strains were assessed using Dunn’s *post hoc* test, with *P* < 0.05 considered statistically significant.

## Supplementary Material

Reviewer comments

## Data Availability

Assemblies were deposited under the BioProject PRJNA1447475, with accession numbers JBXRRT000000000J (UC4308), JBXRRU000000000 (UC4317), JBXRRV000000000 (UC4316) and JBXRRW000000000 (UC4315).

## SUPPLEMENTAL MATERIAL

Tables S1 and S2. spectrum.03352-25-s0001.docx Table S1: The complete set of virulence genes detected with Virulence Finder. Table S2: Summary of genomic regions identified by MuMmer as absent mutant strains relative to the wild type (WT).

OPEN PEER REVIEW reviewer-comments.pdf An accounting of the reviewer comments and feedback.

ASM does not own the copyrights to Supplemental Material that may be linked to, or accessed through, an article. The authors have granted ASM a non-exclusive, world-wide license to publish the Supplemental Material files. Please contact the corresponding author directly for reuse.
