# Supplementary material for: Locus of Adhesion and Autoaggregation (LAA) pathogenicity island genes hes and sisA are involved in virulence and biofilm formation in LEE-negative Shiga toxin-producing Escherichia coli (STEC)
Source: Microbiol Spectr. 2026 Jun 15;14(7):e03352-25. doi: 10.1128/spectrum.03352-25 (PMC13340028; doi:10.1128/spectrum.03352-25)
Supplement: Tables S1 and S2. — Table S1: The complete set of virulence genes detected with Virulence Finder. Table S2: Summary of genomic regions identified by MuMmer as absent mutant strains relative to the wild type (WT). [file spectrum.03352-25-s0001.docx]

**TABLE S1.** The complete set of virulence genes detected with Virulence Finder (Center of Genomic Epidemiology, <https://cge.food.dtu.dk/services/VirulenceFinder/>) for the wild type strain UC4224 and mutant strains. The table includes the name of virulence factor, the position on the genome, the protein function and the reference accession number.

| ***E. coli* strain UC4224 (wild type)** | | | | | |
| --- | --- | --- | --- | --- | --- |
| **Virulence factor** | **Identity** | **Query / Template length** | **Position in contig** | **Protein function** | **Accession number** |
| csgA | 98.9 | 456 / 456 | 4759933..4760388 | curlin major subunit CsgA | CP069646 |
| fdeC | 96.07 | 4254 / 4254 | 3891429..3895682 | intimin-like adhesin FdeC | AP010953 |
| fimH | 100.0 | 489 / 489 | 3481827..3482315 | Type 1 fimbriae | NA |
| gad | 100.0 | 1401 / 1401 | 2541068..2542468 | Glutamate decarboxylase | AP010953 |
| gad | 99.36 | 1401 / 1401 | 259195..260595 | Glutamate decarboxylase | CP000038 |
| hha | 92.22 | 180 / 213 | 1905002..1905181 | hemolysin expression modulator Hha (previous rmoA) | 453331 |
| hlyE | 99.02 | 918 / 918 | 4935118..4936035 | Avian E. coli haemolysin | ECU57430 |
| **hra** | 91.44 | 759 / 792 | 1842328..1843082 | Heat-resistant agglutinin | CP028310 |
| iss | 98.98 | 294 / 294 | 4166610..4166903 | Increased serum survival | CP001509 |
| lpfA | 99.83 | 573 / 573 | 2800504..2801076 | Long polar fimbriae | AP010953 |
| lpfA | 99.83 | 573 / 573 | 2800504..2801076 | Long polar fimbriae | KC207123 |
| nlpI | 99.66 | 885 / 885 | 2195396..2196280 | lipoprotein NlpI precursor | CP000243 |
| ompT | 100.0 | 954 / 954 | 4194798..4195751 | Outer membrane protease (protein protease 7) | AMVC01000243 |
| papC | 100.0 | 2511 / 2511 | 3397513..3400023 | Outer membrane usher P fimbriae | JNPN01000012 |
| **stx1-S.-sonnei-CB7888** | 99.92 | 1227 / 1227 | 712405..713631 | stx1-S.-sonnei-CB7888 | AJ279086 |
| **stx1a-O157-GPU96MM** | 99.92 | 1227 / 1227 | 712405..713631 | stx1a-O157-GPU96MM | AB035142 |
| **stx2a-O22-EBC217** | 100.0 | 1241 / 1241 | 4855779..4857019 | stx2a-O22-EBC217 | AY443054 |
| terC | 99.86 | 714 / 714 | 1677714..1678427 | Tellurium ion resistance protein | CP007491 |
| terC | 99.42 | 1041 / 1041 | 2058904..2059944 | Tellurium ion resistance protein | CYAS01000072 |
| terC | 99.9 | 966 / 966 | 2118278..2119243 | Tellurium ion resistance protein | MG591698 |
| terC | 99.42 | 1041 / 1041 | 2058904..2059944 | Tellurium ion resistance protein | UGDL01000003 |
| yehA | 98.16 | 1033 / 1035 | 943684..944716 | Outer membrane lipoprotein, YHD fimbriael cluster | CP042934 |
| yehB | 97.9 | 2481 / 2481 | 944732..947212 | Usher, YHD fimbriael cluster | CP042934 |
| yehC | 99.41 | 675 / 675 | 947228..947902 | Chaperone, YHD fimbriael cluster | CP042934 |
| yehD | 98.71 | 543 / 543 | 947983..948525 | Major pilin subunit, YHD fimbriael cluster | CP042934 |
| yghJ | 97.66 | 4571 / 4569 | 1936989..1941554 |  | CP043217 |
| **stx1** | 100.0 | 1227 / 1227 | 712405..713631 | S.dysenteriae 3818T | M19437 |
| **stx2** | 100.0 | 1241 / 1241 | 4855779..4857019 | O22 EBC217, variant a | AY443054 |

| ***E. coli* strain UC4315** ***(Δhes*-*sisA)*** | | | | | |
| --- | --- | --- | --- | --- | --- |
| **Virulence factor** | **Identity** | **Query / Template length** | **Position in contig** | **Protein function** | **Accession number** |
| csgA | 98.9 | 456 / 456 | 4757651..4758106 | curlin major subunit CsgA | CP069646 |
| fdeC | 96.07 | 4254 / 4254 | 3889160..3893413 | intimin-like adhesin FdeC | AP010953 |
| fimH | 100.0 | 489 / 489 | 3479551..3480039 | Type 1 fimbriae | NA |
| gad | 100.0 | 1401 / 1401 | 2538824..2540224 | Glutamate decarboxylase | AP010953 |
| gad | 99.36 | 1401 / 1401 | 259188..260588 | Glutamate decarboxylase | CP000038 |
| hha | 92.22 | 180 / 213 | 1902770..1902949 | hemolysin expression modulator Hha (previous rmoA) | 453331 |
| hlyE | 99.02 | 918 / 918 | 4931966..4932883 | Avian E. coli haemolysin | ECU57430 |
| iss | 98.98 | 294 / 294 | 4164342..4164635 | Increased serum survival | CP001509 |
| lpfA | 99.83 | 573 / 573 | 2798260..2798832 | Long polar fimbriae | AP010953 |
| lpfA | 99.83 | 573 / 573 | 2798260..2798832 | Long polar fimbriae | KC207123 |
| nlpI | 99.66 | 885 / 885 | 2193153..2194037 | lipoprotein NlpI precursor | CP000243 |
| ompT | 100.0 | 954 / 954 | 4192521..4193474 | Outer membrane protease (protein protease 7) | AMVC01000243 |
| papC | 100.0 | 2511 / 2511 | 3395241..3397751 | Outer membrane usher P fimbriae | JNPN01000012 |
| **stx1-S.-sonnei-CB7888** | 99.92 | 1227 / 1227 | 712380..713606 | stx1-S.-sonnei-CB7888 | AJ279086 |
| **stx1a-O157-GPU96MM** | 99.92 | 1227 / 1227 | 712380..713606 | stx1a-O157-GPU96MM | AB035142 |
| **stx2a-O22-EBC217** | 100.0 | 1241 / 1241 | 4853497..4854737 | stx2a-O22-EBC217 | AY443054 |
| terC | 99.86 | 714 / 714 | 1678902..1679615 | Tellurium ion resistance protein | CP007491 |
| terC | 99.42 | 1041 / 1041 | 2056665..2057705 | Tellurium ion resistance protein | CYAS01000072 |
| terC | 99.9 | 966 / 966 | 2116035..2117000 | Tellurium ion resistance protein | MG591698 |
| terC | 99.42 | 1041 / 1041 | 2056665..2057705 | Tellurium ion resistance protein | UGDL01000003 |
| yehA | 98.16 | 1033 / 1035 | 943659..944691 | Outer membrane lipoprotein, YHD fimbriael cluster | CP042934 |
| yehB | 97.9 | 2481 / 2481 | 944707..947187 | Usher, YHD fimbriael cluster | CP042934 |
| yehC | 99.41 | 675 / 675 | 947203..947877 | Chaperone, YHD fimbriael cluster | CP042934 |
| yehD | 98.71 | 543 / 543 | 947958..948500 | Major pilin subunit, YHD fimbriael cluster | CP042934 |
| yghJ | 97.66 | 4571 / 4569 | 1934753..1939318 | | CP043217 |
| **stx1** | 100.0 | 1227 / 1227 | 712380..713606 | S.dysenteriae 3818T | M19437 |
| **stx2** | 100.0 | 1241 / 1241 | 4853497..4854737 | O22 EBC217, variant a | AY443054 |

| ***E. coli* strain UC4316 (*Δstx1; Δhes*-*sisA)*** | | | | | |
| --- | --- | --- | --- | --- | --- |
| **Virulence factor** | **Identity** | **Query / Template length** | **Position in contig** | **Protein function** | **Accession number** |
| csgA | 98.9 | 456 / 456 | 4756504..4756959 | curlin major subunit CsgA | CP069646 |
| fdeC | 96.07 | 4254 / 4254 | 3888013..3892266 | intimin-like adhesin FdeC | AP010953 |
| fimH | 100.0 | 489 / 489 | 3478403..3478891 | Type 1 fimbriae | NA |
| gad | 100.0 | 1401 / 1401 | 2350748..2352148 | Glutamate decarboxylase | AP010953 |
| gad | 99.36 | 1401 / 1401 | 259188..260588 | Glutamate decarboxylase | CP000038 |
| hha | 92.22 | 180 / 213 | 2846300..2846479 | hemolysin expression modulator Hha (previous rmoA) | 453331 |
| hlyE | 99.02 | 918 / 918 | 4930820..4931737 | Avian E. coli haemolysin | ECU57430 |
| iss | 98.98 | 294 / 294 | 4163195..4163488 | Increased serum survival | CP001509 |
| lpfA | 99.83 | 573 / 573 | 2610184..2610756 | Long polar fimbriae | AP010953 |
| lpfA | 99.83 | 573 / 573 | 2610184..2610756 | Long polar fimbriae | KC207123 |
| nlpI | 99.66 | 885 / 885 | 2005077..2005961 | lipoprotein NlpI precursor | CP000243 |
| ompT | 100.0 | 954 / 954 | 4191374..4192327 | Outer membrane protease (protein protease 7) | AMVC01000243 |
| papC | 100.0 | 2511 / 2511 | 3394093..3396603 | Outer membrane usher P fimbriae | JNPN01000012 |
| **stx2a-O22-EBC217** | 100.0 | 1241 / 1241 | 4852351..4853591 | stx2a-O22-EBC217 | AY443054 |
| terC | 99.86 | 714 / 714 | 1677754..1678467 | Tellurium ion resistance protein | CP007491 |
| terC | 99.42 | 1041 / 1041 | 2878472..2879512 | Tellurium ion resistance protein | CYAS01000072 |
| terC | 99.9 | 966 / 966 | 1927959..1928924 | Tellurium ion resistance protein | MG591698 |
| terC | 99.42 | 1041 / 1041 | 2878472..2879512 | Tellurium ion resistance protein | UGDL01000003 |
| yehA | 98.16 | 1033 / 1035 | 943781..944813 | Outer membrane lipoprotein, YHD fimbriael cluster | CP042934 |
| yehB | 97.9 | 2481 / 2481 | 944829..947309 | Usher, YHD fimbriael cluster | CP042934 |
| yehC | 99.41 | 675 / 675 | 947325..947999 | Chaperone, YHD fimbriael cluster | CP042934 |
| yehD | 98.71 | 543 / 543 | 948080..948622 | Major pilin subunit, YHD fimbriael cluster | CP042934 |
| yghJ | 97.66 | 4571 / 4569 | 2809931..2814496 |  | CP043217 |
| **stx2** | 100.0 | 1241 / 1241 | 4852351..4853591 | O22 EBC217, variant a | AY443054 |

| ***E. coli* strain UC4317 (*Δstx2; Δhes*-*sisA)*** | | | | | |
| --- | --- | --- | --- | --- | --- |
| **Virulence factor** | **Identity** | **Query / Template length** | **Position in contig** | **Protein function** | **Accession number** |
| csgA | 98.9 | 456 / 456 | 78697..79152 | curlin major subunit CsgA | CP069646 |
| fdeC | 96.07 | 4254 / 4254 | 943390..947643 | intimin-like adhesin FdeC | AP010953 |
| fimH | 100.0 | 489 / 489 | 1356764..1357252 | Type 1 fimbriae | NA |
| gad | 100.0 | 1401 / 1401 | 2296579..2297979 | Glutamate decarboxylase | AP010953 |
| gad | 99.36 | 1401 / 1401 | 4574897..4576297 | Glutamate decarboxylase | CP000038 |
| hha | 92.22 | 180 / 213 | 2933854..2934033 | hemolysin expression modulator Hha (previous rmoA) | 453331 |
| hlyE | 99.02 | 918 / 918 | 4946783..4947700 | Avian E. coli haemolysin | ECU57430 |
| iss | 98.98 | 294 / 294 | 672168..672461 | Increased serum survival | CP001509 |
| lpfA | 99.83 | 573 / 573 | 2037971..2038543 | Long polar fimbriae | AP010953 |
| lpfA | 99.83 | 573 / 573 | 2037971..2038543 | Long polar fimbriae | KC207123 |
| nlpI | 99.66 | 885 / 885 | 2642766..2643650 | lipoprotein NlpI precursor | CP000243 |
| ompT | 100.0 | 954 / 954 | 643329..644282 | Outer membrane protease (protein protease 7) | AMVC01000243 |
| papC | 100.0 | 2511 / 2511 | 1439052..1441562 | Outer membrane usher P fimbriae | JNPN01000012 |
| **stx1-S.-sonnei-CB7888** | 99.92 | 1227 / 1227 | 4121905..4123131 | stx1-S.-sonnei-CB7888 | AJ279086 |
| **stx1a-O157-GPU96MM** | 99.92 | 1227 / 1227 | 4121905..4123131 | stx1a-O157-GPU96MM | AB035142 |
| terC | 99.86 | 714 / 714 | 3157188..3157901 | Tellurium ion resistance protein | CP007491 |
| terC | 99.42 | 1041 / 1041 | 2779098..2780138 | Tellurium ion resistance protein | CYAS01000072 |
| terC | 99.9 | 966 / 966 | 2719803..2720768 | Tellurium ion resistance protein | MG591698 |
| terC | 99.42 | 1041 / 1041 | 2779098..2780138 | Tellurium ion resistance protein | UGDL01000003 |
| yehA | 98.16 | 1033 / 1035 | 3890820..3891852 | Outer membrane lipoprotein, YHD fimbriael cluster | CP042934 |
| yehB | 97.9 | 2481 / 2481 | 3888324..3890804 | Usher, YHD fimbriael cluster | CP042934 |
| yehC | 99.41 | 675 / 675 | 3887634..3888308 | Chaperone, YHD fimbriael cluster | CP042934 |
| yehD | 98.71 | 543 / 543 | 3887011..3887553 | Major pilin subunit, YHD fimbriael cluster | CP042934 |
| yghJ | 97.66 | 4571 / 4569 | 2897485..2902050 | | CP043217 |
| **stx1** | 100.0 | 1227 / 1227 | 4121905..4123131 | S.dysenteriae 3818T | M19437 |

| ***E. coli* strain UC4308 (*Δstx1; Δstx2; Δhes*-*sisA)*** | | | | | |
| --- | --- | --- | --- | --- | --- |
| **Virulence factor** | **Identity** | **Query / Template length** | **Position in contig** | **Protein function** | **Accession number** |
| csgA | 98.9 | 456 / 456 | 2866867..2867322 | curlin major subunit CsgA | CP069646 |
| fdeC | 96.07 | 4254 / 4254 | 3731560..3735813 | intimin-like adhesin FdeC | AP010953 |
| fimH | 100.0 | 489 / 489 | 4144934..4145422 | Type 1 fimbriae | NA |
| gad | 100.0 | 1401 / 1401 | 225668..227068 | Glutamate decarboxylase | AP010953 |
| gad | 99.36 | 1401 / 1401 | 2504181..2505581 | Glutamate decarboxylase | CP000038 |
| hha | 92.22 | 180 / 213 | 862943..863122 | hemolysin expression modulator Hha (previous rmoA) | 453331 |
| hlyE | 99.02 | 918 / 918 | 72090..73007 | Avian E. coli haemolysin | ECU57430 |
| iss | 98.98 | 294 / 294 | 3460338..3460631 | Increased serum survival | CP001509 |
| lpfA | 99.83 | 573 / 573 | 4827420..4827992 | Long polar fimbriae | AP010953 |
| lpfA | 99.83 | 573 / 573 | 4827420..4827992 | Long polar fimbriae | KC207123 |
| nlpI | 99.66 | 885 / 885 | 571855..572739 | lipoprotein NlpI precursor | CP000243 |
| ompT | 100.0 | 954 / 954 | 3431499..3432452 | Outer membrane protease (protein protease 7) | AMVC01000243 |
| papC | 100.0 | 2511 / 2511 | 4227222..4229732 | Outer membrane usher P fimbriae | JNPN01000012 |
| terC | 99.86 | 714 / 714 | 1086277..1086990 | Tellurium ion resistance protein | CP007491 |
| terC | 99.42 | 1041 / 1041 | 708187..709227 | Tellurium ion resistance protein | CYAS01000072 |
| terC | 99.9 | 966 / 966 | 648892..649857 | Tellurium ion resistance protein | MG591698 |
| terC | 99.42 | 1041 / 1041 | 708187..709227 | Tellurium ion resistance protein | UGDL01000003 |
| yehA | 98.16 | 1033 / 1035 | 1819978..1821010 | Outer membrane lipoprotein, YHD fimbriael cluster | CP042934 |
| yehB | 97.9 | 2481 / 2481 | 1817482..1819962 | Usher, YHD fimbriael cluster | CP042934 |
| yehC | 99.41 | 675 / 675 | 1816792..1817466 | Chaperone, YHD fimbriael cluster | CP042934 |
| yehD | 98.71 | 543 / 543 | 1816169..1816711 | Major pilin subunit, YHD fimbriael cluster | CP042934 |
| yghJ | 97.66 | 4571 / 4569 | 826574..831139 | | CP043217 |

**TABLE S2.** Summary of genomic regions identified by MuMmer as absent mutant strains relative to the wild type (WT). For each gap detected in the WT genome, the table reports the corresponding genomic features in the mutant strain that overlap or fall within these regions. Listed are the WT gap coordinates and length, along with locus tags, predicted gene products, genomic coordinates in the mutant genome, and strand orientation.

| **strain** | **WT_gap_start** | **WT_gap_end** | **locus_tag** | **product** | **feature_start** | **feature_end** | **strand** |
| --- | --- | --- | --- | --- | --- | --- | --- |
| UC4315 | 1839515 | 1844278 | N4S17_08945 | tetratricopeptide repeat protein | 1839242 | 1840285 | + |
| UC4315 | 1839515 | 1844278 | N4S17_08950 | Phosphoethanolamine transferase | 1840644 | 1842146 | - |
| UC4315 | 1839515 | 1844278 | N4S17_08955 | outer membrane beta-barrel protein | 1842328 | 1843068 | - |
| UC4315 | 1839515 | 1844278 | N4S17_08960 | Signal transduction histidine-protein kinase AtoS | 1843501 | 1845327 | + |
|  | | | | | | | |
| UC4316 | 712429 | 713606 | N4S17_03545 | Shiga toxin subunit B | 712405 | 712674 | - |
| UC4316 | 712429 | 713606 | N4S17_03550 | Shiga toxin subunit A | 712684 | 713631 | - |
| UC4316 | 1839515 | 1844278 | N4S17_08945 | tetratricopeptide repeat protein | 1839242 | 1840285 | + |
| UC4316 | 1839515 | 1844278 | N4S17_08950 | Phosphoethanolamine transferase | 1840644 | 1842146 | - |
| UC4316 | 1839515 | 1844278 | N4S17_08955 | outer membrane beta-barrel protein | 1842328 | 1843068 | - |
| UC4316 | 1839515 | 1844278 | N4S17_08960 | Signal transduction histidine-protein kinase AtoS | 1843501 | 1845327 | + |
|  | | | | | | | |
| UC4317 | 1512881 | 1512940 | UC4224_01454 | Transcriptional repressor MprA | 1512539 | 1513069 | + |
| UC4317 | 1839515 | 1844278 | N4S17_08945 | tetratricopeptide repeat protein | 1839242 | 1840285 | + |
| UC4317 | 1839515 | 1844278 | N4S17_08950 | Phosphoethanolamine transferase | 1840644 | 1842146 | - |
| UC4317 | 1839515 | 1844278 | N4S17_08955 | outer membrane beta-barrel protein | 1842328 | 1843068 | - |
| UC4317 | 1839515 | 1844278 | N4S17_08960 | Signal transduction histidine-protein kinase AtoS | 1843501 | 1845327 | + |
| UC4317 | 4855803 | 4856994 | N4S17_23455 | Shiga toxin subunit A | 4855779 | 4856738 | + |
| UC4317 | 4855803 | 4856994 | N4S17_23460 | Shiga toxin subunit B | 4856750 | 4857019 | + |
|  | | | | | | | |
| UC4308 | 712429 | 713606 | UC4224_00702 | Shiga toxin subunit A | 712684 | 713631 | - |
| UC4308 | 712429 | 713606 | UC4224_00701 | Shiga toxin subunit B | 712405 | 712674 | - |
| UC4308 | 1839515 | 1844278 | N4S17_08945 | tetratricopeptide repeat protein | 1839242 | 1840285 | + |
| UC4308 | 1839515 | 1844278 | N4S17_08950 | Phosphoethanolamine transferase | 1840644 | 1842146 | - |
| UC4308 | 1839515 | 1844278 | N4S17_08955 | outer membrane beta-barrel protein | 1842328 | 1843068 | - |
| UC4308 | 1839515 | 1844278 | N4S17_08960 | Signal transduction histidine-protein kinase AtoS | 1843501 | 1845327 | + |
| UC4308 | 4856049 | 4856739 | UC4224_04635 | Shiga toxin subunit A | 4855779 | 4856738 | + |
