# Supplementary material for: Locus of Adhesion and Autoaggregation (LAA) pathogenicity island genes hes and sisA are involved in virulence and biofilm formation in LEE-negative Shiga toxin-producing Escherichia coli (STEC)
Source: Microbiol Spectr. 2026 Jun 15;14(7):e03352-25. doi: 10.1128/spectrum.03352-25 (PMC13340028; doi:10.1128/spectrum.03352-25)
Supplement: Reviewer comments [file reviewer-comments.pdf]

AMERICAN SOCIETY FOR MICROBIOLOGY | Microbiology Spectrum

# Locus of Adhesion and Autoaggregation (LAA) pathogenicity island genes *hes* and *sisA* are involved in virulence and biofilm formation in LEE-negative Shiga-toxin producing *Escherichia coli* (STEC)

Giovanni Milani, Claudia Cortimiglia, Gabriele Bellotti, Franco Lucchini, and Pier Sandro Cocconcelli

*Corresponding Author(s): Claudia Cortimiglia, Universita Cattolica del Sacro Cuore - Campus di Piacenza e Cremona*

**Review Timeline:**

| | |
|---|---|
| Submission Date: | October 31, 2025 |
| Editorial Decision: | January 13, 2026 |
| Revision Received: | April 14, 2026 |
| Accepted: | April 22, 2026 |

*Editor: Gillian Tarr*

*Reviewer(s): Disclosure of reviewer identity is with reference to reviewer comments included in decision letter(s). The following individuals involved in review of your submission have agreed to reveal their identity: Rahat Zaheer (Reviewer #1); Adriana Bentancor (Reviewer #2)*

## Transaction Report:

(Note: With the exception of the correction of typographical or spelling errors that could be a source of ambiguity, letters and reports are not edited. The original formatting of letters and referee reports may not be reflected in this compilation.)

**DOI: https://doi.org/10.1128/spectrum.03352-25**

Re: Spectrum03352-25 (**Locus of Adhesion and Autoaggregation (LAA) pathogenicity island genes*hes* and *sisA* enhance virulence and biofilm formation in LEE-negative Shiga-toxin producing*Escherichia coli* (STEC)**)

Dear Dr. Claudia Cortimiglia:

Thank you for the privilege of reviewing your work. Below you will find my comments, instructions from the Spectrum editorial office, and the reviewer comments.

Editor's Comments
1. Always capitalize Shiga, as it is the name of the discovering scientist.
2. Genes should always be italicized. If referring to the toxins themselves, they should be un-italicized and capitalized (e.g. Stx1).
3. Please indicate the serotype of the strain studied.
4. Please review the full manuscript for grammatical errors.
5. Lines 72-73: These are serogroups, not serotypes, and a mention of the dominant disease-causing serogroups is remiss without mention of O157.

Instructions
Please return the manuscript within 60 days; if you cannot complete the modification within this time period, please contact me. If you do not wish to modify the manuscript and prefer to submit it to another journal, notify me immediately so that the manuscript may be formally withdrawn from consideration by Spectrum.

**Revision Guidelines**
To submit your modified manuscript, log into the submission site at https://spectrum.msubmit.net/cgi-bin/main.plex. Go to Author Tasks and click the appropriate manuscript title to begin. The information you entered when you first submitted the paper will be displayed; update this as necessary. Note the following requirements:

• Upload point-by-point responses to the issues raised by the reviewers in a file named "Response to Reviewers," NOT in your cover letter.
• Upload a compare copy of the manuscript (without figures) as a "Marked-Up Manuscript" file.
• Upload a clean .DOC/.DOCX version of the revised manuscript and remove the previous version.
• Each figure must be uploaded as a separate, editable, high-resolution file (TIFF or EPS preferred), and any multipanel figures must be assembled into one file.
• Any supplemental material intended for posting by ASM should be uploaded with their legends separate from the main manuscript. You can combine all supplemental material into one file (preferred) or split it into a maximum of 10 files with all associated legends included.

For complete guidelines on revision requirements, see our Submission and Review Process webpage. Submission of a paper that does not conform to guidelines may delay acceptance of your manuscript.

**Data availability:** ASM policy requires that data be available to the public upon online posting of the article, so please verify all links to sequence records, if present, and make sure that each number retrieves the full record of the data. If a new accession number is not linked or a link is broken, provide Spectrum production staff with the correct URL for the record. If the accession numbers for new data are not publicly accessible before the expected online posting of the article, publication may be delayed; please contact production staff (Spectrum@asmusa.org) immediately with the expected release date.

**Publication Fees:** For information on publication fees and which article types are subject to charges, visit ourwebsite. If your manuscript is accepted for publication and any fees apply, you will be contacted separately about payment during the production process; please follow the instructions in that e-mail. Arrangements for payment must be made before your article is published.

**ASM Membership:** Corresponding authors may join or renew ASM membership to obtain discounts on publication fees. Need to upgrade your membership level? Please contact Customer Service at Service@asmusa.org.

The ASM Journals program strives for constant improvement in our submission and publication process. Please tell us how we can improve your experience by taking this quick Author Survey.

Thank you for submitting your paper to Spectrum.

Sincerely,
Gillian Tarr
Editor
Microbiology Spectrum

Reviewer #1 (Comments for the Author):

This manuscript by Milani et al. addresses an important and timely topic in the field of STEC research, focusing on LEE-negative strains and the role of the LAA pathogenicity island. The study is well-designed, employing a combination of genetic engineering, in vivo infection modeling with Galleria mellonella, biofilm assays, and adhesion studies. The use of targeted deletions in hes and sisA, combined with stx gene deletions, provides a clear framework for evaluating the contribution of these genes to virulence and colonization-associated traits. The integration of multiple experimental approaches strengthens the conclusions, particularly by linking gene deletion to both reduced mortality in larvae and decreased biofilm formation and adhesion in vitro. The manuscript is comprehensive in its literature coverage, placing the work in the context of emerging LEE-negative STEC strains, and highlights the relevance of alternative virulence factors beyond Shiga toxins. Overall, the study contributes novel insights into the genetic determinants of STEC pathogenicity and could inform future surveillance and risk assessment strategies.

However, despite its strengths, the study has several limitations (see below) that should be acknowledged:
- The experiments were conducted using a single LAA-positive STEC strain, which limits the generalizability of the findings across different STEC backgrounds.
- This study lacks gene complementation experiments to confirm that the observed phenotypes are specifically due to the hes-sisA deletion rather than polar effects on adjacent genes or unintended secondary mutations. Inclusion of complementation assays would strengthen the causal link between these genes and the observed reductions in virulence, adhesion, and biofilm formation.
- The use of Galleria mellonella as the sole in vivo infection model, while convenient and ethically acceptable, cannot fully replicate mammalian host-pathogen interactions, particularly due to the lack of adaptive immunity and physiological differences. The deletion of a ~5 kb region may have polar effects on adjacent genes or regulatory elements, which could influence observed phenotypes beyond the intended targets.
- Some statements in the Abstract and Discussion overstate causality or extrapolate to human infection; these should be toned down to reflect model limitations.
- Additionally, the manuscript has minor issues with English phrasing and grammar, including occasional typos, inconsistent italicization of gene names, and occasional informal or non-scientific wording (e.g., "less invisible to the immune system"). Revising these aspects will improve clarity, readability, and precision of scientific communication.
- Finally, the manuscript could benefit from further discussion of how these findings might be validated in additional strains and complementary models, to strengthen translational relevance.

Please see attached manuscript file with some specific comments and edits.

Reviewer #2 (Comments for the Author):

Locus of Adhesion and Autoaggregation (LAA) pathogenicity island genes hes and sisA enhance virulence and biofilm formation in LEE-negative Shiga-toxin producing Escherichia coli (STEC)
This work enables the identification of a set of genes within the LAA pathogenicity island that contribute to the virulence of LEE-negative STEC strains. The objective is clearly defined and would allow discrimination of LEE-negative STEC isolates with a higher molecular risk profile.
Previous studies have already identified hes as the most relevant gene within LAA as a marker of this pathogenicity island and have proposed it as a virulence marker for LEE-negative strains.
In the present study, two genes, hes and sisA, are considered key determinants of virulence risk in a strain, based on the need to identify new molecular risk markers for STEC isolates. Although the role of sisA has been described in Shigella, there is no specific information regarding its contribution in STEC or its role within the LAA island.
Major comments
The study was conducted using a strain isolated from cheese, whose origin could be bovine, buffalo, ovine, or caprine (this information is not specified), which does not guarantee pathogenic potential in humans. At a minimum, this information should be included in the Materials and Methods section and incorporated into the Discussion.
The hes gene was originally proposed as an LAA marker based on studies of LEE-negative strains associated with human disease, in which its presence was linked to clinical pathogenic potential. In the present work, however, the analysed strain originates from raw-milk cheese rather than from a human isolate. It cannot be automatically assumed that the role of hes and of the hes-sisA region is equivalent in human and non-human contexts.
With regard to the use of Caco-2 cells, these are of human origin. It would therefore be interesting to explicitly propose future studies using cell lines of bovine origin.
Regarding the conclusions about LAA, the manuscript draws broader conclusions than are supported by the data. The study evaluated the deletion of a specific fragment of LAA but did not perform complementation. The results demonstrate that this region has a significant impact on virulence, biofilm formation, and adhesion. However, the effects observed are extrapolated to the entire LAA island, despite the fact that LAA comprises additional modules that were not evaluated in this work. Moreover,

given that the strain is of animal origin, its regulatory and functional context may differ from that of a human isolate. Therefore, rather than concluding that "LAA" is a key determinant of virulence, the conclusions should specifically refer to the role of this particular LAA region.

The critical role of hes in biofilm formation and interactions with cell lines has been reported previously. The immediate question, therefore, concerns the specific contribution of sisA. Can any differential effect be attributed to this gene? At the current stage, the results neither allow this to be confirmed nor ruled out, but this issue should be discussed explicitly.

No discriminatory information regarding the specific effect of sisA has been presented.

With respect to the original results obtained using Galleria mellonella, there are also some concerns. Given that Stx2 is approximately 100-fold more potent than Stx1, why is the $LD_{50}$ of the UC4177 ($stx1^{+}$) strain higher than that of the UC4176 ($stx2^{+}$) strain? This raises the question of whether G. mellonella is an appropriate model in this context. More importantly, regarding the experimental design, the studies for each strain were performed in triplicate, but the number of larvae per treatment group is not specified. This information is critical: if "triplicate" refers to three larvae per strain, the rigour of the in vivo model would be insufficient. One of the advantages of G. mellonella as an in vivo model is precisely that the use of a larger number of individuals per treatment increases the robustness of the results. This information should be clearly provided in order to assess the validity of the Kaplan-Meier survival analyses.

The presentation of survival percentages in the graphs is difficult to interpret because the bars overlap or are not clearly visible. It may be necessary to provide additional material or to split the figure into separate panels: one for toxin mutants and another for hes-sisA mutants with their respective controls, selecting a single concentration that allows visualisation of the additive effect of hes-sisA on virulence. The remaining concentrations could be included as supplementary material. Since it is reported that all larvae die at a dose of $10^{4}$, including this graph does not appear to add meaningful information.

Minor comments

1. The writing requires careful revision. For example, "agn 4" is mentioned instead of agn43. Similarly, toxin genes are inconsistently written in lowercase. Toxin subtypes are mentioned based on a bibliographic citation (6) that does not correspond to the statement made. The protocol for the in vivo G. mellonella assay is not described in reference 16, nor in reference 37. Throughout the manuscript, the use of italics (including in the reference list) should be carefully checked and standardised.
2. The identification of strain UC4224 as the wild type would benefit from a clearer and more hierarchical presentation. Grouping mutant strains according to the type of modification-first, mutants affecting only Shiga toxins; second, the mutant with deletion of the hes-sisA fragment of LAA; and finally, the combined mutants-would greatly improve the flow and clarity of the text.
3. Datsenko and Wanner (2000) should be explicitly cited. The mutagenesis strategy employed is the lambda Red recombination system, originally described by Datsenko and Wanner, which has since become a canonical method in Escherichia coli genetics.

# Locus of Adhesion and Autoaggregation (LAA) pathogenicity island genes *hes* and *sisA* enhance virulence and biofilm formation in LEE-negative Shiga-toxin producing *Escherichia coli* (STEC)

Giovanni Milani, Claudia Cortimiglia*, Franco Lucchini, Pier Sandro Cocconcelli

*Department for Sustainable Food Process (DiSTAS), Università Cattolica del Sacro Cuore, Piacenza/Cremona, Italy*

**Corresponding author:** *Claudia Cortimiglia, claudia.cortimiglia@unicatt.it

**Keywords:** STEC, LAA PAI, virulence, adhesion, biofilm, *hes*, *sisA*.

**Running title**

**Involvement of *hes* and *sisA* genes in STEC virulence**

**ABSTRACT**

Shiga toxin-producing *Escherichia coli* (STEC) is a major foodborne pathogen, capable of causing severe diseases such as Hemolytic Uremic Syndrome (HUS), particularly in children. While the Locus of Enterocyte Effacement (LEE) has long been associated with STEC virulence, an increasing number of LEE-negative strains are being linked to human infections. Some strains harbor the Locus of Adhesion and Autoaggregation (LAA), a pathogenicity island comprising genes implicated in adhesion and biofilm formation. In this study, we investigated the role of two LAA-associated genes, *hes* and *sisA*, in the virulence of the LEE-negative STEC strain UC4224. Using lambda red recombineering, we generated mutant strains with deletions in *stx1*, *stx2*, and/or the *hes*-*sisA* region. *Galleria mellonella* larvae were employed as an *in vivo* infection model to evaluate pathogenicity. Mutants were also assessed for the ability to produce biofilm and adhere to epithelial cell lines. Our results revealed that deletion of *hes-sisA* significantly decreased larval mortality. Deletion of *hes-sisA* abolished biofilm formation, and significantly reduced adhesion to Caco-2 intestinal cells. These findings demonstrate that *hes* and *sisA* genes contribute significantly to STEC virulence, biofilm formation, and epithelial adhesion, supporting their role as key elements of the LAA PAI. This work highlights the importance of LAA in the pathogenicity of LEE-negative STEC strains and suggests that alternative virulence factors beyond Shiga toxins are critical to disease progression. Further studies are warranted to explore the functional roles of these and other genes within the LAA region across different STEC backgrounds.

**IMPORTANCE (150 words)**

Shiga toxin-producing *Escherichia coli* (STEC) is the third most common cause of gastrointestinal illness in Europe. Cattle act as asymptomatic reservoirs, transmitting infection to humans mainly through contaminated raw milk and meat. Several outbreaks linked to raw milk products have been reported in recent years, prompting EU Rapid Alert System notifications, including in 2024. These infections represent a major public health and economic concern. In 2019, EFSA recommended enhanced surveillance and a broader assessment of STEC pathogenicity, emphasizing virulence factors beyond Shiga toxins and intimin. This study investigates a specific genomic region within a pathogenicity island to elucidate its role in virulence

and biofilm formation. The findings highlight additional molecular markers that may contribute to STEC pathogenic potential. Incorporating these markers into molecular screening and surveillance can improve the identification of high-risk STEC clones, supporting more accurate risk assessment and targeted public health interventions.

## INTRODUCTION

Shiga toxin-producing *Escherichia coli* (STEC) is a significant cause of foodborne illness, leading to conditions ranging from mild gastroenteritis to haemorrhagic colitis and severe Hemolytic Uremic Syndrome (HUS), especially in children below 5 years (1). This pathogen has been associated with several outbreaks mainly linked to the consumption of raw dairy and meat products, and vegetable-based foods. According to the last European Union One Health Zoonoses report, the number of STEC found in food and isolated from humans increased by 130 % in 2023 compared to 2020, raising from 4,446 to 10,217 cases, with a growth of outbreaks related to dairy products (2). This upward trend highlights STEC as an emerging public health concern and underscores the need to better understand its environmental persistence, survival in food, and virulence mechanisms (3).

Historically, STEC strains have been classified into serotypes, with the "top six" (O26, O45, O103, O111, O121 and O145) linked to higher pathogenicity. However, the genomic plasticity of *E. coli* makes serotyping alone an unreliable predictor of clinical outcomes (4). STEC pathogenicity is known to be primarily associated with Shiga toxins (5), classified into two types, namely Stx1 and Stx2, of which different subtypes have been described (Stx1a-Stxe and Stx2a-Stx2l) (6). Although the most severe clinical cases have been mainly associated with some of these Stx variants (6), the outcome of the infection depends on other virulence factors and host-related factors such as the health status and the age of human host (7, 8). Some highly pathogenic STEC strains harbour LEE, a pathogenicity island which plays a crucial role in enabling adherence to and damage of host intestinal epithelial cells (9, 10). The compromising of intestinal epithelial cells is further aggravated by the presence of Shiga toxins, leading to major complications of symptoms (11).

Although LEE-positive STEC strains have been identified as the cause of the most severe forms of gastrointestinal disorders and HUS, in some cases these diseases have been linked to LEE-negative strains

(12). In particular, European Food Safety Authority (EFSA) reported an increase in the number of *eae*-negative STEC strains isolated from animals, food and humans in 2023 compared to 2022 (2).

In 2017, Montero et al. characterized a novel 80 kb-long pathogenicity island in LEE-negative STEC strains, designated as the Locus of Adhesion and Autoaggregation (LAA) (13). A large-scale genomic analysis performed on LEE-negative STEC strains isolated from humans, animals and food, revealed that among pathogenicity islands (PAIs) in LEE negative STEC strains, LAA PAI was the most prevalent and most associated with *stx* variants (*stx*1a, *stx*2a, *stx*2c and *stx*2d) mainly linked to the most severe outcomes of infection (14). Several genes including *hes*, *iha*, *lesP*, *pagC*, *tpsA*, *tpsB*, and *agn4* can be recognized as markers for the four modules that make up the LAA, which can exist as a whole or partial structure (13). Some of the genes harboured by this region were studied for their involvement in adhesion to epithelial cells and colonization in a mouse model, hypothesising that they may make these strains particularly resistant in food and confer pathogenicity traits (14). In particular, *hes* gene was suggested to be the marker of LAA PAI, as it had been detected in all strains with a complete LAA island and in most LEE-negative strains (14). Recently, this gene was also demonstrated to be involved in the formation of biofilm in a STEC O91 strain (15). In the first LAA module, near *hes*, another gene called *sisA* has been described with a role in the immunomodulation. *SisA* gene is the homolog of *Shigella flexneri shiA,* a gene encoded by SHI-2 pathogencity island, linked to the induction of inflammation (16, 17). In particular, s*hiA* was suggested to mediate the suppression of the innate response during *Shigella* infections, without reducing the invasion and cytotoxicity in the mouse lung model (17). The *shiA* homolog in *Escherichia coli* strains has been subjected to very few studies (18, 19). The study of Lloyd and colleagues (19) revealed the presence of two s*hiA* homologue genes, *sisA* and *sisB*, with differing percentages of identity (83.3% and 96.8%, respectively). *SisA* was found to be involved in suppressing the host's immune response but contributed to a lesser extent to reducing the inflammatory response. They also observed that *sisA* was typical of extraintestinal pathogenic *Escherichia coli* (ExPEC) strains, while *sisB* was found more in the enteric strains.

Despite ongoing efforts, few and sometimes conflicting data on the pathogenicity of LAA is available. Expanding our understanding of the biological role of this genomic region is of paramount importance.

In our previous work (20), starting from a LAA-positive STEC strain isolated from raw milk cheese, we generated mutant strains lacking *stx1*, *stx2* or both genes. Pathogenicity was evaluated using the *Galleria*

*mellonella* infection model, which revealed that the inactivation of Shiga toxins did not completely abolish the virulent potential of the strains, suggesting the involvement of additional factors responsible for the strain pathogenicity. In particular, we hypothesized that the persistence of pathogenicity could be linked to genes located within the LAA PAI. Based on this, we selected a specific portion of the LAA region to investigate its role in virulence and environmental survival. The aims of this study were: i) to use a genome engineering strategy to delete a portion of the first LAA PAI module, creating mutants with different combinations of deletions in *stx1*, *stx*2 and/or the selected LAA segment; ii) to assess the pathogenicity of these modified strains in *Galleria mellonella* larvae and compare their $LD_{50}$ values to evaluate the contribution of the targeted LAA region to virulence; iii) to evaluate the involvement of the region of LAA PAI in adhesion to Caco-2 cell lines and biofilm on food manufacturing surfaces.

## Results

### *Construction of hes and sisA null mutants*

To investigate the role of *hes* and *sisA* genes, located within the LAA region, in pathogenicity and biofilm development, STEC strain UC4224, and its derivatives UC4176 (UC4224*Δstx1::kan*, Kan$^R$ ), UC4177 (UC4224*Δstx2::cat*, Cm$^R$ ) and UC4178 (UC4224*Δstx1::kan Δstx2::cat*, Kan$^R$ Cm$^R$ ) were subjected to a 5485 bp deletion. Using the lambda red recombineering, *hes*-*sisA* deleted strains were successfully generated: UC4315 (UC4224 *Δ hes*-*sisA ::Tet*, Tet$^R$ ), UC4316 (UC4224*Δstx1::kan*, *Δ hes*-*sisA ::Tet,* Kan$^R$ Tet$^R$), UC4317 (UC4224*Δstx2::cat*, *Δ hes*-*sisA ::Tet,* Cm$^R$ Tet$^R$ ) and UC4308 (UC4224*Δstx1::kan Δstx2::cat*, *Δ hes*-*sisA ::Tet,* Kan$^R$ Cm$^R$ Tet$^R$) (Table 1). The substitution of this region with the tetracycline resistance cassette (*TetA*) was confirmed by PCR analysis and Sanger sequencing.

### *The involvement of hes and sisA genes in the pathogenicity against Galleria mellonella*

In this study, we evaluated the virulence of strains derived from a LAA-positive STEC strain in which *stx1, stx2* and of a portion of the LAA PAI were deleted. The mortality rate was assessed by injecting *G. mellonella* larvae with bacterial suspensions ranging from $10^1$ to $10^7$ CFU/10µl of the 4 mutant strains, their

respective LAA-positive counterparts and *E. coli* BL21 as a negative control. Untreated larvae and those exposed to 0.1 M PBS and different doses of *E. coli* BL21 showed no significant mortality. Mortality rates observed in larvae treated with UC4224, UC4176 (*Δstx1*), UC4177 (*Δstx2*) and UC4178 (*Δstx1*, *Δstx2*) were consistent with the finding reported by Milani et al. (20).

The Kaplan-Meier survival analysis (Fig. 1) was performed on the four lowest doses ($10^1$ to $10^4$ CFU/10µl), since at concentrations above 4 Log CFU/10 µL the mortality rates for the UC4315 (*Δhes-sisA)*, UC4316 (*Δstx1, Δhes-sisA*) and UC4317 (*Δstx2, Δhes-sisA*) mutants were 100% after 24 hours. When the two mutants UC4316 (*Δstx1, Δhes-sisA*) and UC4317 (*Δstx2, Δhes-sisA*) were tested, the obtained mortality rates were significantly lower than UC4315 (*Δhes-sisA)* ($p < 0.05$) for the four lowest doses injected. UC4317 (*Δstx2, Δhes-sisA*) exhibited reduced pathogenicity compared to UC4316 (*Δstx1, Δhes-sisA*) ($p < 0.05$) exclusively at $10^4$ CFU/10 µL. Significant reductions in mortality rates were also found comparing the LAA PAI null mutants with the respective originating strains. In particular, UC4315 (*Δhes-sisA*) was significantly less pathogenic than UC4224 (wild type) ($p < 0.05$) when injected with $10^1$ to $10^3$ CFU/10µl. On the contrary, when *G. mellonella* larvae were injected with $10^4$ CFU/10 µl of UC4315(*Δhes-sisA*), the mortality rate was 100% after 24 hours. Furthermore, when comparing the pathogenicity of UC4316 (*Δstx1, Δhes-sisA*) and UC4317 (*Δstx2, Δhes-sisA*) with their respective parents UC4176 (*Δstx1*) and UC4177 (*Δstx2*), a significant reduction in the mortality rate of *G. mellonella* was observed when treated with concentrations from $10^1$ to$10^4$ CFU/10 µl. Finally, UC4308 (*Δstx1, Δstx2, hes-sisA*) was revealed to be significantly ($p < 0.05$) less pathogenic than its originating strain UC4178 (*Δstx1, Δstx2*), when injected with loads of $10^2$ CFU/10 µL to $10^6$ CFU/10 µL. When treated with concentrations higher than $10^6$ CFU/10 µL, UC4308 (*Δstx1, Δstx2, hes-sisA*) showed 100% mortality after 24 hours. Additionally, UC4308 (*Δstx1, Δstx2, hes-sisA*) was demonstrated to be significantly lower pathogenic than UC4315 (*Δhes-sisA)*, when inoculated at $10^1$ to $10^5$ CFU/10µl. Moreover, we observed that UC4308 (*Δstx1, Δstx2, hes-sisA*) was significantly less pathogenic than UC4316 (*Δstx1, Δhes-sisA*) and UC4317 (*Δstx2, Δhes-sisA*) when the injected doses were $10^3$-$10^4$ CFU/10µl. Interestingly, at concentrations of $10^3$ CFU/10 µL or lower, no significant differences in mortality were observed between the UC4308 mutant and the non-pathogenic *E. coli* BL21($p < 0.05$).

Then, the probit regression model was employed to determine the $LD_{50}$ after 24 hours for all tested strains. Overall, we observed an increase in the $LD_{50}$, as additional virulence factors were deleted. Among the

strains, UC4224 exhibited the lowest $LD_{50}$, consistent with previous findings (20) (Fig 2). The UC4315 mutant (*Δhes-sisA*), which still harbored both intact *stx* operons, showed a $LD_{50}$ of $1.4\times10^2$ CFU/10 µL, significantly higher than that of the wild type UC4224 ($p < 0.05$). No statistically significant differences were observed in the $LD_{50}$ values of the mutants lacking *stx1* (UC4176), *stx2* (UC4177), or the *hes-sisA* region (UC4315).

The deletion of a portion of the LAA PAI from UC4176 (*Δstx1*) to generate UC4316 (*Δstx1, Δhes-sisA*) resulted in a significant increase in $LD_{50}$, reaching $9.4\times10^2$ CFU/10 µL. Furthermore, strain UC4317 (*Δstx2, Δhes-sisA*) exhibited an $LD_{50}$ of $7.7\times10^3$ CFU/10 µL, representing two logarithmic orders increase compared to UC4177(*Δstx2*), highlighting a statistically significant difference ($p < 0.05$) in the number of cells causing the death of larvae. Notably, the $LD_{50}$ of UC4317 (*Δstx2, Δhes-sisA*) was also significantly higher than that of UC4178 (*Δstx1*, *Δstx2*), which lacks both *stx* genes, suggesting a cumulative impact of the additional LAA deletion. No statistically significant difference in $LD_{50}$ was observed between UC4316 (*Δstx1, Δhes-sisA*) and UC4317 (*Δstx2, Δhes-sisA*).

The concomitant absence of both *stx* and *hes-sisA* genes in UC4308 (*Δstx1, Δstx2, Δhes-sisA*) resulted in a further increase in $LD_{50}$ to $1.4\times10^5$ CFU/10 µL, making it significantly less pathogenic than all the other strains, excluding UC4317 (*Δstx2, Δhes-sisA*). Although the difference between $LD_{50}$ values of UC4308 (*Δstx1, Δstx2, Δhes-sisA*) and UC4317 (*Δstx2, Δhes-sisA*) was not significant, it is noteworthy that the pathogenicity of UC4308 (*Δstx1, Δstx2, Δhes-sisA*) was reduced to more than one Log CFU/10 µL.

These *in vivo* results indicated higher survival rates for larvae treated with mutants in the 5kb region of LAA PAI than those exposed to their respective strains harbouring *sisA-hes* genes. This is particularly evident for UC4308, where deletions of the *stx1*, *stx2*, *hes*, and *sisA* genes resulted in a marked reduction in pathogenicity.

**FIG 1**. Kaplan-Meier survival curve of Galleria mellonella larvae injected with different loads: $10^1$ CFU/10µl (A), $10^2$ CFU/10µl (B), $10^3$ CFU/10µl (C) and $10^4$ CFU/10µl (D). Larval survival was monitored for 24h, 48h and 72 h. The results were obtained from triplicates of the same experiment. Larvae were also inoculated with negative controls, namely PBS and E. coli BL21.

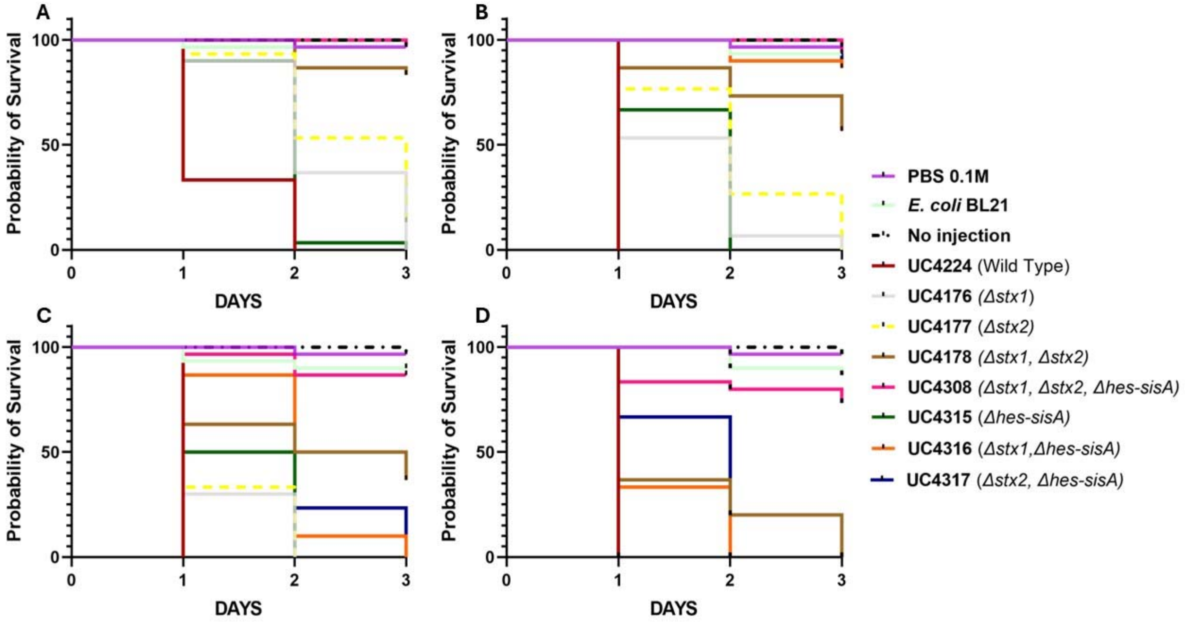


**FIG 2.** $LD_{50}$ values calculated for the wild type STEC UC4224 and its derivative mutants in the pathogenicity assay in *Galleria mellonella* larvae. Bars represent the mean values, and error bars indicate the 95% confidence intervals. Different letters above the bars indicate statistically significant differences between strains ($p < 0.05$).

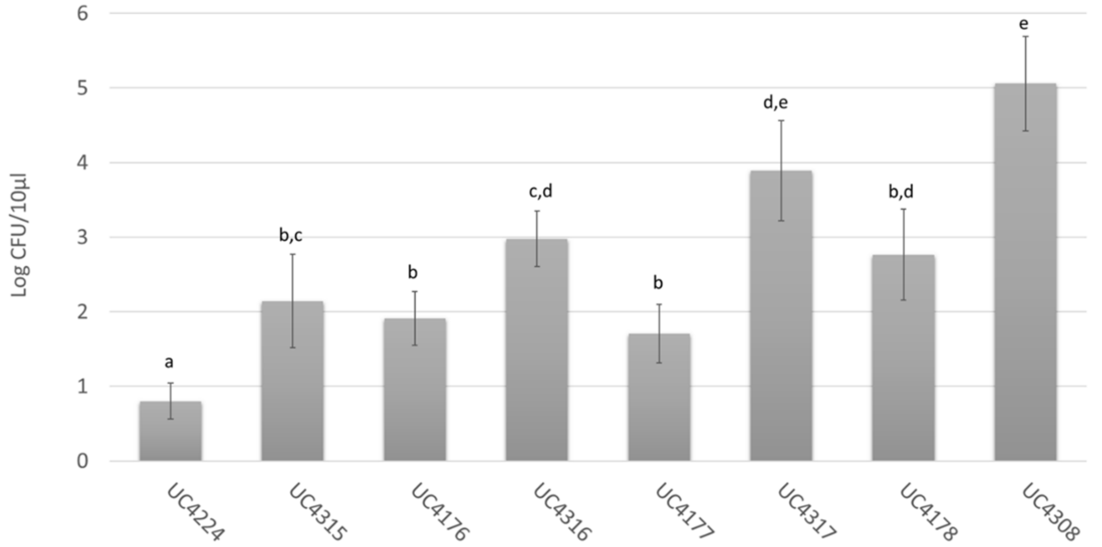


| | UC4224 | UC4315 | UC4176 | UC4316 | UC4177 | UC4317 | UC4178 | UC4308 |
|---|---|---|---|---|---|---|---|---|
| *stx1* | + | + | - | - | + | + | - | - |
| *stx2* | + | + | + | + | - | - | - | - |
| *hes-sisA* | + | - | + | - | + | - | + | - |

***Involvement of hes and sisA genes in biofilm production***

The ability of the wild type STEC UC4224 strain and the six related mutants to produce biofilm on polystyrene and milk/stainless steel interface, representing the typical condition of dairy production, was assessed. No significant differences ($p$ <0.05) in biofilm production on both types of surfaces were observed for UC4224, and the single and double mutants in shiga-toxin genes. In contrast, UC4315 (*Δhes-sisA)*, UC4316 (*Δstx1, Δhes-sisA*), UC4317 (*Δstx2, Δhes-sisA*), and UC4308 (*Δstx1, Δstx2, Δhes-sisA*) strains, lacking the *hes* and *sisA* genes, exhibited a complete loss of this ability (Fig. 3). The biofilm production levels in these mutants were comparable to those of the negative control, and significantly lower than those of strains retaining an intact LAA PAI region.

**FIG 3**. Biofilm formation of the wild type UC4224 and related mutants. The blue series (A) is related to the formation of biofilm on plastics, while the grey series (B) is related to the biofilm production on stainless steel. The quantification of biofilm was assessed by $OD_{595}$. Error bars indicate standard deviation.

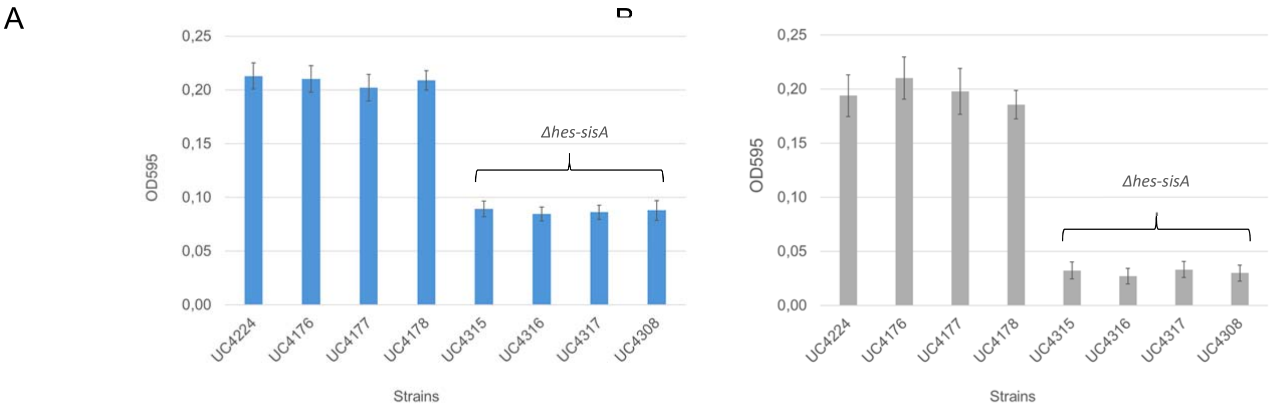


***Contribution of hes and sisA on Caco-2 cell adhesion***

The role of *hes* and *sisA* genes in Caco-2 cell adhesion was assessed for the STEC strain UC4224 and its seven mutants (Fig. 4A). The highest adhesion levels were observed for *L. rhamnosus* GG (ATCC 53103), with statistically significant differences observed ($p < 0.05$). A significant reduction in adhesion ability ($p < 0.05$) was noted in mutants lacking *hes* and *sisA* genes (UC4315, UC4316 and UC4308) compared to strains UC4224, UC4176 (*Δstx1*), UC4177(*Δstx2*), and UC4178 (*Δstx1*, *Δstx2*), which retain these genes (Fig. 4B). No significant differences were observed among strains with an intact LAA-PAI region. Similarly, no statistically significant differences in adhesion were found among UC4315(*Δhes-sisA)*, UC4316 (*Δstx1, Δhes-sisA*), UC4317(*Δstx2, Δhes-sisA*), and UC4308 (*Δstx1, Δstx2, Δhes-sisA*) and *L. delbrueckii* subsp. *lactis* (DSM 20072).

**FIG 4.** Adhesion of *Escherichia coli* UC4224 and related mutants to Caco-2 cells. A) Representative images illustrate the adhesion of the wild-type strain UC4224 and its mutants carrying deletions in *stx1*, *stx2*, *hes-*

*sisA*, or their combinations. Reference strains *L. rhamnosus* GG and *L. delbrueckii* subsp. *lactis* were included. B) The graph reports the mean ± standard deviation of the number of adherent bacteria per nucleus for each strain. For each strain, the assay was performed in triplicates, considering 15 randomly considered microscopic fields. *L. delbrueckii* subsp. *lactis* (DSM 20072) and *L. rhamnosus* GG (ATCC 53103) were used as positive and negative controls, respectively. Error bars indicate standard deviation between triplicates. Letters represent the significant differences between means ($p < 0.05$).

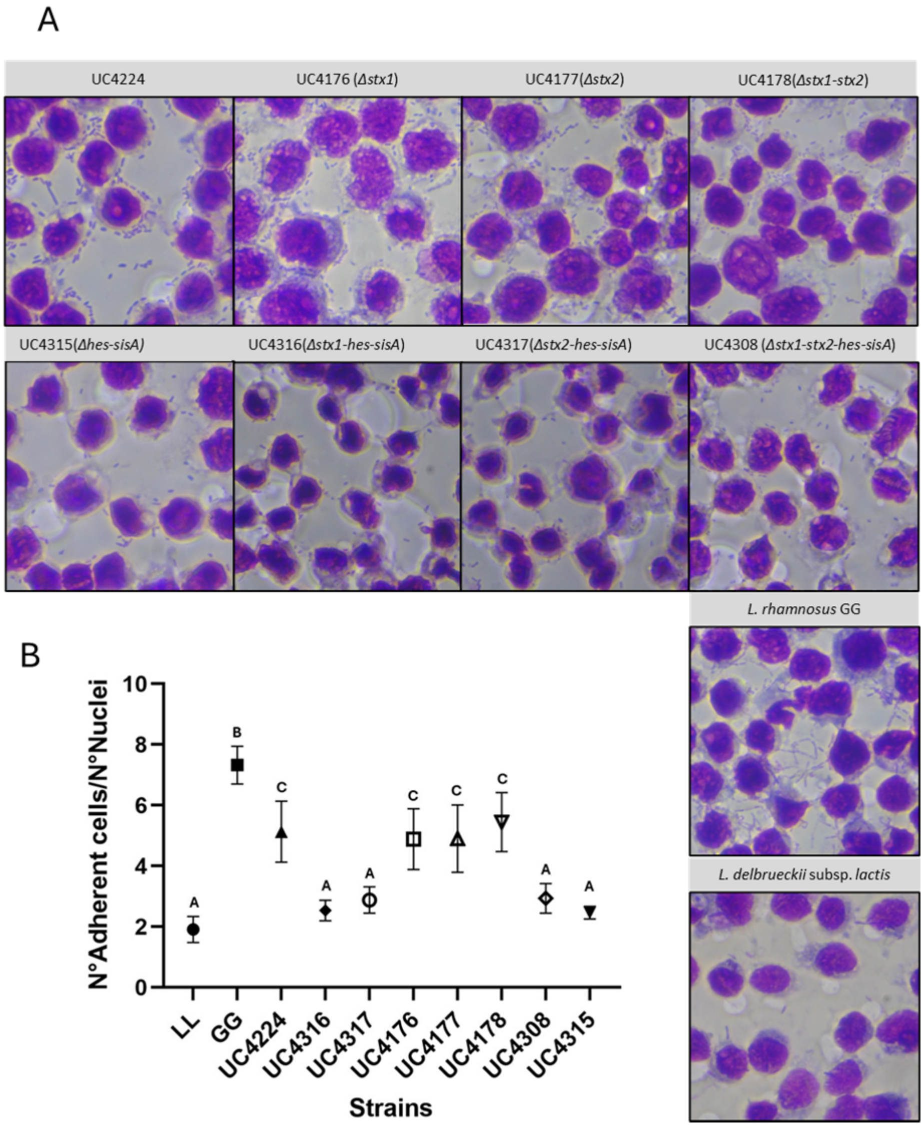

**Discussion**

LEE-negative STEC strains represent an important group of pathogenic bacteria that lack a genomic region typically associated with the virulence of enteropathogenic and enterohemorrhagic *E. coli* (21). Albeit the absence of the LEE locus, these strains are able to employ alternative virulence mechanisms to adhere to and invade host tissues. Recently, there has been increasing scientific interest in the investigation of LEE-negative STEC strains due to their association with severe diseases (22, 23). Indeed, the number of LEE-negative STEC strains isolated from patients with HUS in Japan (12) and in some parts of Europe has been increasing (23, 24).

Some of these strains lacked LEE locus, but were found to harbour the LAA pathogenicity island, which contains genes encoding adhesion factors that contribute to their pathogenicity (13). The LAA PAI region was named by Montero and colleagues as it contains several novel and known virulence factors associated with bacterial adhesion and autoaggregation properties (13). These genes are crucial for both tight adhesions to host cells and the structured architecture of biofilms. Biofilm development represents an adaptive response that allows STEC strains to endure unfavorable conditions, thereby enhancing their survival in different environments such as food and increasing the risk of host infection (25). This capability varies across and within strains and is influenced by factors beyond just the microbial strain involved. While the LEE locus has been deeply characterized, the function of the 80 genes on the LAA pathogenicity island has yet to be elucidated. Indeed, understanding the genetic backgrounds and virulence profiles of these LEE-negative strains is critical for developing effective diagnostic and therapeutic strategies to mitigate the risks associated with infections (26). Here, for the first time, we investigated the role of two genes, *hes* and *sisA*, in the virulence of a LAA-positive STEC strain isolated from a semi-hard raw milk cheese using an *in vivo* model,

such as *Galleria mellonella*, thereby advancing our understanding of a region within the LAA PAI that has only recently been described.

Building upon previously constructed *stx*-deleted derivatives (20), we successfully generated additional mutant strains carrying targeted deletion within a 5 kb region of the LAA PAI. These strains were then used to evaluate the contribution of these genes to virulence. Firstly, the pathogenicity assessment in *G. mellonella* demonstrated that their deletion caused a significant reduction in mortality rate following inoculation with the double mutants UC4316 (*Δstx1, Δhes-sisA*) or UC4317 (*Δstx2, Δhes-sisA*) compared with UC4315 (*Δhes-sisA*). This observation is consistent with the current knowledge about the link between pathogenicity and presence of *stx* genes (27, 28). Additionally, UC4316 (*Δstx1, Δhes-sisA*) and UC4317 (*Δstx2, Δhes-sisA*) caused lower mortality rates than their respective parental strains UC4176 (*Δstx1*) and UC4177 (*Δstx2*), which retain *hes* and *sisA* genes. These data were confirmed by the reduction of the mortality rate of the triple mutant UC4308 respect to the UC4178 strain, lacking *stx* genes and harboring the LAA PAI. Moreover, UC4308 was revealed to be less virulent in respect to UC4316 (*Δstx1, Δhes-sisA*) and UC4317 (*Δstx2, Δhes-sisA*). Overall, these results suggested that this region within the I module of LAA PAI significantly contributed to the virulence of the UC4224 strain.

The hypothesis is further supported by the analysis of $LD_{50}$ values. As shown in Fig. 2, the $LD_{50}$ value increased significantly by one Log unit for strains lacking *hes* and *sisA*, compared to the parental strains. A progressive increase in $LD_{50}$ was observed as additional virulence-associated genes were deleted. Specifically, the $LD_{50}$ of double mutants was one Log higher than that of the single mutants, while the triple mutant exhibited an $LD_{50}$ value one Log higher than the double mutants. These findings confirm a cumulative effect of gene deletions on the attenuation of virulence, suggesting that *hes* and *sisA* play a significant role in the pathogenic potential of this STEC strain.

Previously, *hes* was investigated by Montero et al. (13) for the involvement in the agglutination of sheep erythrocytes, in the autoaggregation and in the biofilm formation. The involvement of *hes* in the biofilm formation was further confirmed by another study, in which deletion of the gene resulted in reduced biofilm production and alterations in its structure (15). The ability to mediate adherence and form biofilm can be considered as a virulence factor because facilitates the colonization and resistance to host immune defenses,

other than persistence in hostile environments. Indeed, the deletion of *hes* may have attenuate the colonization potential, further lowering the pathogenic process. In addition, we deleted *sisA*, which was suggested to be involved in the downregulation of inflammatory responses in the host, as previously described in *Shigella* species. By potentially downregulating inflammatory responses and influencing innate immune cell functions, microbes are able to avoid immune detection and promote the survival. The simultaneous lack of *hes* and *sisA* may have made strains UC4315 (*Δhes-sisA*), UC4316 (*Δstx1, Δhes-sisA*), UC4317 (*Δstx2, Δhes-sisA*) and UC4178 (*Δstx1, Δstx2, Δhes-sisA*) less suited to adhesion and less "invisible" to the immune system of *Galleria mellonella*, thus making them less pathogenic.

We further investigate the effect of *hes* deletion on the biofilm formation. Consistent with findings from other studies using different strains, we observed that deletion of a region including *hes* led to a marked reduction in biofilm formation on both stainless steel and polystyrene surfaces (Fig. 3). This finding is particularly relevant in the context of recent trends. The increasing number of STEC infections reported by EFSA along with the growing prevalence of LEE-negative strains isolated from different sources may reflect selective pressure acting on STEC population. These pressures could be promoting the genomic reorganization, enhancing the adaptability and environmental persistence of these pathogens (8, 29, 30). Pathogenic *E. coli* strains exhibiting hybrid virulence profiles are being reported with increasing frequency. This trend suggests that evolutionary pressures are actively driving the emergence of diverse combinations of virulence factors, primarily through horizontal gene transfer, resulting in the formation of distinct virulence-associated genomic islands (31, 32). Notably, these events have also involved LAA-positive strains, as previously reported by other studies (14).

Adhesion of bacterial strains to intestinal epithelial cells is a key factor in the colonization and persistence of STEC within the human gut (33, 34). Wild-type and mutant strains carrying *hes* and *sisA* genes exhibited significantly greater adhesion to Caco-2 cells compared to strains lacking these genes, supporting their role in mediating attachment (Fig. 4). In contrast, previous studies using HEp-2 cells reported more variable outcomes, potentially due to strain-specific expression patterns or cell-type differences (35). Such discrepancies could be justified by the fact that additional genes may contribute to adhesion, and that this trait is likely influenced by both physiological and environmental conditions. Indeed, the expression of

adhesins appears to be a coordinated process that depends on the specific strain background as well as the surrounding environment (36).

While our findings provide new insights, some limitations need to be acknowledged to properly frame their interpretation. These results were obtained from a single LAA PAI-positive STEC strain, but additional data with different STEC strains carrying different combinations of LAA PAI modules are required to confirm these findings. Moreover, although the *G. mellonella* infection model provides a convenient and ethically acceptable system to assess bacterial virulence, some limitations should be acknowledged. Indeed, it lacks an adaptive immune system, and thus cannot fully reproduce the complex host–pathogen interactions occurring in mammals. Moreover, physiological differences, including gut environment, body temperature regulation, and tissue organization, may influence bacterial colonization and the expression of virulence factors. While larvae can be maintained at 37 °C, prolonged exposure to this temperature may affect their immune competence and survival, potentially introducing variability. Finally, heterogeneity in larval age, size, and rearing conditions can affect reproducibility across experiments (37).

In conclusion, our study provides significant insights into the pathogenic potential of LEE-negative STEC strains, particularly those harboring the LAA pathogenicity island. The findings indicate that even in the absence of the conventional LEE locus, these strains can employ alternative virulence mechanisms, underscored by the critical roles of the *hes* and *sisA* genes, which enhance adherence and pathogenicity. The reduced virulence and increased $LD_{50}$ values observed in the double and triple mutants highlight the cumulative impact of gene deletions on pathogenicity, emphasizing the importance of the LAA-positive strains in the overall virulence profile.

Furthermore, our observations on adhesion to intestinal epithelial cells reinforce the essential role of these genes in promoting colonization and persistence of LEE-negative STEC in the gastrointestinal tract. In addition, we demonstrated a correlation between the absence of a specific segment of the LAA pathogenicity island and enhanced biofilm formation at the milk/stainless steel interface, a feature particularly relevant to the persistence of these strains in dairy processing environments.

This work expands our understanding of the genetic and functional diversity of pathogenic mechanisms employed by STEC strains and emphasizes the need for further research to uncover the breadth of virulence

factors encoded by the LAA PAI. Such knowledge is crucial for developing effective diagnostic and therapeutic strategies to manage infections associated with these emerging pathogens. Given the increasing isolation of LEE-negative strains from severe disease cases, deepening our understanding of their virulence determinants is critical for public health and clinical interventions. Further studies involving additional LAA-positive STEC strains are needed to validate and extend these findings.

## Materials and methods

### *Bacterial Strains, Plasmids and Media*

Bacterial strains used in this study are listed and described in Table 1. UC4224 STEC strain, isolated from semi-hard raw milk cheese, was grown in Luria-Bertani (LB) broth (Sigma-Aldrich). The mutated strains UC4176 (UC4224*Δstx1*), UC4177 (UC4224*Δstx2*) and UC4178 (UC4224*Δstx1-Δstx2*) were grown in LB broth supplemented with chloramphenicol (Cm) (Sigma-Aldrich) (50 µg/ml), kanamycin (Kan) (Sigma-Aldrich) (50 µg/ml), and tetracycline (Sigma-Aldrich) (3.5–10 µg/ml) when needed (20) .

The *E. coli* strain DH5α, used for the propagation and purification of plasmids, was grown in LB broth supplemented with Amp (Amp) (Sigma-Aldrich) (100 µg/mL). Lactobacillaceae were propagated in De Man–Rogosa–Sharp (MRS) (OXOID) under anaerobic conditions at 37°C.

**Table 1**. Bacterial strains and plasmids used in this study.

| **Strain** | **Relevant genotype, phenotype** | **Reference/Source** |
|---|---|---|
| *E. coli* UC4224 | STEC parental strain | (20) |
| *E. coli* UC4315 | UC4224 *Δhes-sisA*::Tet, Tet$^R$ | This study |
| *E. coli* UC4176 | UC4224*Δstx1*::kan, Kan$^R$ | (20) |
| *E. coli* UC4316 | UC4224*Δstx1*::kan, *Δhes-sisA*::Tet, Kan$^R$ Tet$^R$ | This study |
| *E. coli* UC4177 | UC4224*Δstx2*::cat, Cm$^R$ | (20) |
| *E. coli* UC4317 | UC4224*Δstx2*::cat, *Δhes-sisA*::Tet, Cm$^R$ Tet$^R$ | This study |
| *E. coli* UC4178 | UC4224*Δstx1*::kan *Δstx2*::cat, Kan$^R$ Cm$^R$ | (20) |
| *E. coli* UC4308 | UC4224*Δstx1*::kan, *Δstx2*::cat, *Δhes-sisA*::Tet, Kan$^R$ Cm$^R$ Tet$^R$ | This study |
| *E. coli* BL21 | Negative control in pathogenicity assay | DSM 102053 |
| *Lactobacillus delbrueckii* subsp. | Negative control in Caco2 Adhesion assay | DSM 20072 |

| | | |
|---|---|---|
| *lactis* | | |
| *Lacticaseibacillus rhamnosus* – GG | Positive control in Caco2 Adhesion assay | ATCC 53103 |
| **Plasmids** | | |
| pSIM6 | Plasmid expressing Lambda red recombination genes below the control of CI857 repressor, $Amp^R$ (Ts) | (38) |
| pACYC184 | Template plasmid for the amplification of FRT-Tet-FRT amplicon, $Tet^R$ $Cm^R$ | (39) |

Amp – ampicillin, Tet – Tetracycline, Kan – Kanamycin, Cm – chloramphenicol, superscripts "R" and "S" represent resistance and sensitivity

***Construction the amplimer containing the tetracycline resistance cassette for the partial deletion of LAA PAI***

A 5,485 bp region of the LAA pathogenicity island (PAI), encompassing the *hes* and *sisA* genes, was chosen as the target to be substituted by the tetracycline cassette. The strategy to obtain the fragment for the recombineering was previously described (20, 39). Briefly, the tetracycline resistance cassette was firstly amplified from plasmid pACYC184 using primers TC-5 and TC-3 (Table 1). Then, primers LAA-UP/Tet-5'LAA and Tet-3'LAA/LAA-Down were designed for the amplification of a 373 bp upstream region (Homologous arm UP) and 350 bp of LAA PAI downstream region (Homologous arm DOWN), respectively. The tetracycline resistance cassette was then amplified with primers Tet-5'LAA and Tet-3'LAA, to obtain the cassette with upstream and downstream homologous regions. Ultimately, primers LAA-UP and LAA-Down were used to obtain the final fragment of 2066 bp. All amplified fragments were loaded on electrophoresis gel and purified with NucleoSpin™ Gel and PCR Clean-up (Macherey-Nagel). After the purification of the final fragment, a second round of PCR was conducted using the same primer pair to increase the amount of amplicon.

For the primer design, Primer3web version 4.1.0 (https://primer3.ut.ee/) was used, and the specificity was checked against the fragment to be amplified, and against the STEC UC4224 genome (Table 2).

All the PCR reactions were performed using Phusion Flash High-Fidelity PCR Master Mix (ThermoFisher Scientific) following the manufacturer's instruction.

**Table 2**. Oligonucleotides used in this study.

| Primer | Sequence (3’-5’) | Reference |
|---|---|---|
| Tc-5 | TCAGCCCCATACGATATAAG | [17] |
| Tc-3 | TGGAGTGGTGAATCCGTTAG | |
| LAA-UP | GCAACGTTAATCCCGGACAA | This study |
| Tet-5’LAA | CTTATATCGTATGGGGCTGACCAACTGCCAGCCCTTTAAG | |
| Tet-3’LAA | CTAACGGATTCACCACTCCACGCGGACACTTAACGATCTG | This study |
| LAA-DOWN | GGTTTCTTTGCGGGCAGTTA | |

***Transformation with plasmids pSIM6, recombineering and replacement confirmation***

The strategy for the transformation and recombineering is described in detail in a previous work [16,35]. Recombinants were identified after 1 to 2 days of incubation at 37 °C. The resulting mutants were UC4315 (UC4224 *Δhes-sisA ::Tet*, Tet$^R$), UC4316 (UC4224*Δstx1::kan*, *Δhes-sisA::Tet,* Kan$^R$ Tet$^R$), UC4317 (UC4224*Δstx2::cat*, *Δhes-sisA::Tet,* Cm$^R$ Tet$^R$), and UC4308 (UC4224*Δstx1::kan Δstx2::cat*, *Δhes-sisA::Tet,* Kan$^R$ Cm$^R$ Tet$^R$) (Table 1).

The obtained recombinants were cured as previously described (20). The correct substitution was confirmed by a locus-specific PCR using the primers LAA-UP and LAA-DOWN (Table 2), which provide amplicons differing in size between the parental strain and the respective recombinant. PCR products were sequenced by commercial facility (Eurofins Genomics, Italy) using Sanger technology.

***Pathogenicity assessment in Galleria mellonella***

The pathogenicity of new mutants UC4315 (*Δhes*-*sisA*), UC4316 *Δstx1, Δhes*-*sisA* , UC4317 (*Δstx2, Δhes*-*sisA*), UC4308 (*Δstx1, Δstx2, Δhes*-*sisA*) was tested *in vivo* using *Galleria mellonella* larvae with the protocol previously described [16]. The strains UC4224 (wild type), UC4176 (*Δstx1*), UC4177 (*Δstx2*) and UC4178 (*Δstx1, Δstx2*) were also tested, other than BL21 and PBS 0.1M as negative controls. Larvae were injected with 10 µl of tenfold dilutions (from $10^1$ to $10^7$ CFU for each larvae). The survival rate of each strain at each injection doses was determined using Kaplan–Meier survival curve provided by GraphPad Prism (Survival curve 8.4.3 (686)). The significant differences in probability of survival among all strains were generated using Logrank tests ($p < 0.05$), while the $LD_{50}$ values were calculated using Probit Analysis, according to the methodology of Finney (1971) with a 95% confidence limit (40).

***Biofilm assay***

The biofilm-forming ability of wild-type STEC UC4224 and its mutants was initially evaluated using 96-well polystyrene microtiter plates (Sarstedt, Nümbrecht, Germany) in triplicate, following a previously described protocol (15) with minor modifications. Overnight LB cultures grown at 37 °C were adjusted to an optical density (OD) at 620 nm of 0.5, corresponding to approximately $2 \times 10^8$ CFU/ml. A 10 µl aliquot of each culture was inoculated into 190 µl of reconstituted skim milk (Oxoid) and incubated at 37 °C for 24 h. Plates were then further incubated for 48 h at 37 °C, after which they were washed three times with sterile distilled water, fixed with 100% methanol, and stained with 1% crystal violet. Excess stain was removed by repeated washing with distilled water, and biofilm formation was quantified by measuring the OD595 using a Multiskan EX microplate reader (Thermo Electron Corporation, Waltham, MA, USA).

Subsequently, the adhesion capacity on stainless steel (SS) spheres was evaluated following Bassi et al. 2017, with minor changes (41). Ten sterile SS spheres (5 mm diameter) were placed in a 50 ml tube containing 10 ml of reconstituted skim milk (OXOID), inoculated with 50 µl of an overnight culture ($1x10^9$ CFU/ml) for each strain. After 24 h incubation at 37°C, SS spheres were removed from the milk, washed three times with distilled water to eliminate unattached cells from the biofilm and stained with 0.1% crystal violet solution for 15 min. Excess of crystal violet solution was removed by washing twice with distilled water. Spheres were subsequently resuspended with 3 ml of ethanol/acetone solution (80/20, v/v) and

vortexed for 3 minutes to detach biofilm cells. Therefore, the OD at 595 nm of the resulting solution was determined. Three independent replicates of each strain were carried out for each strain.

Statistical analysis was conducted using one-way ANOVA followed by Tukey's HSD test to identify significant differences in biofilm formation across strains.

***Caco-2 cell lines preparation and adhesion assay***

Enterocyte-like Caco-2 cells p25 (ATCC HTB 37) were grown in Dulbecco's modified Eagle's minimal essential medium (DMEM) (VWR International), supplemented with 10% (v/v) heat inactivated fetal bovine serum (Euroclone, Pero, Italy), 2 mM L-glutamine (Euroclone), and gentamycin (50 µg/mL) (Euroclone). Incubation of cell lines was carried out at 37 °C in a 95% (v/v) atmosphere with 5% (v/v) $CO_2$. After reaching the confluence, the concentration of Caco-2 monolayer was evaluated by trypsinizing the cells for 10 min at 37°C and counting them in a hemocytometer. For adhesion assay, Caco-2 cell lines were seeded on glass coverslips located in 24-well tissue culture plates (Sarstedt). An amount of 500 µl containing $5\times10^4$ cells were transferred to each well and incubated at 37 °C in a 95% (v/v) atmosphere with 5% (v/v) $CO_2$ for 2 weeks. The culture medium was replaced every 48 h.

Adhesion experiments with STEC UC4224 and its seven mutant strains to epithelial Caco-2 cells were carried out as previously described by Tuomola 1998 and Jacobsen 1999 with some modifications (42). Briefly, twelve hours prior to the assay, the culture medium of the Caco-2 cells was replaced with fresh antibiotic-free DMEM. The two week-old monolayers were washed twice with phosphate-buffered saline (PBS, pH 7.4). Overnight bacterial cultures of the wild type strain and mutant strains were adjusted to an $OD_{600}$ of 0.5 in PBS, corresponding to approximately 2 x $10^8$ CFU/ml. *Lacticaseibacillus rhamnosus* GG (ATCC 53103) and *Lactobacillus delbrueckii* subsp. *lactis* (DSM 20072) were included as positive and negative controls, respectively. A total of 300 µl of each bacterial suspension was added to the Caco-2 monolayers and incubated at 37°C for 1 hour. The monolayers were washed four times with PBS, fixed with methanol at room temperature for 10 minutes and then stained with a 1:20 dilution of Giemsa stain solution (Merck, Darmstadt, Germany) for 30 minutes. Slides were washed, air-dried, and examined under oil immersion microscopy at ×100 magnification. Each bacterial strain was tested in triplicate, and bacterial adhesion was quantified by counting adherent cells in 15 randomly selected microscopic fields per slide.

Bacterial adhesion was normalized to the number of host cell nuclei per field. Statistical differences between strains were assessed using Dunn's post hoc test, with $p < 0.05$ considered statistically significant.

## Author contributions

**Giovanni Milani**: Conceptualization, Data Curation, Formal Analysis, Investigation, Methodology, Validation, Visualization, Writing – Original Draft Preparation; **Claudia Cortimiglia**: Methodology

Validation, Writing – Original Draft Preparation, Writing – Review & Editing; **Franco Lucchini**: Data Curation, Methodology, Validation, Writing – Review & Editing; **Pier Sandro Cocconcelli**: Conceptualization, Project Administration, Resources, Supervision, Writing – Review & Editing.

## Declaration of interest statement

The authors report there are no competing interests to declare.

## References

1. Etcheverría AI, Padola NL. 2013. Shiga toxin-producing *Escherichia coli*: Factors involved in virulence and cattle colonization. Virulence 4:366–372.

2. EFSA and ECDC. The European Union One Health 2023 Zoonoses report https://doi.org/10.2903/j.efsa.2024.9106.

3. Vogeleer P, Tremblay YDN, Mafu AA, Jacques M, Harel J. 2014. Life on the outside: role of biofilms in environmental persistence of Shiga-toxin producing Escherichia coli. Front Microbiol 5.

4. EFSA BIOHAZ Panel, Koutsoumanis K, Allende A, Alvarez-Ordóñez A, Bover-Cid S, Chemaly M, Davies R, De Cesare A, Herman L, Hilbert F, Lindqvist R, Nauta M, Peixe L, Ru G, Simmons M, Skandamis P, Suffredini E, Jenkins C, Monteiro Pires S, Morabito S, Niskanen T, Scheutz F, da Silva Felício MT, Messens W, Bolton D. 2020. Pathogenicity assessment of Shiga toxin-producing Escherichia coli (STEC) and the public health risk posed by contamination of food with STEC. EFS2 18.

5. Herold S, Karch H, Schmidt H. 2004. Shiga toxin-encoding bacteriophages – genomes in motion. International Journal of Medical Microbiology 294:115–121.

6. FAO, WHO. 2018. Shiga toxin-producing Escherichia coli (STEC) and food: attribution, characterization, and monitoring.

7. Yan X, Fratamico PM, Bono JL, Baranzoni GM, Chen C-Y. 2015. Genome sequencing and comparative genomics provides insights on the evolutionary dynamics and pathogenic potential of different H-serotypes of Shiga toxin-producing Escherichia coli O104. BMC Microbiol 15:83.

8. Coombes BK, Wickham ME, Mascarenhas M, Gruenheid S, Finlay BB, Karmali MA. 2008. Molecular Analysis as an Aid To Assess the Public Health Risk of Non-O157 Shiga Toxin-Producing *Escherichia coli* Strains. Appl Environ Microbiol 74:2153–2160.

9. Franzin FM, Sircili MP. 2015. Locus of Enterocyte Effacement: A Pathogenicity Island Involved in the Virulence of Enteropathogenic and Enterohemorragic *Escherichia coli* Subjected to a Complex Network of Gene Regulation. BioMed Research International 2015:1–10.

10. Schmidt MA. LEEways: tales of EPEC, ATEC and EHEC.

11. Stevens MP, Frankel GM. 2014. The Locus of Enterocyte Effacement and Associated Virulence Factors of Enterohemorrhagic *Escherichia coli*. Microbiol Spectr 2:2.4.16.

12. Kubomura A, Lee K, Ohnishi M, Iyoda S, Akeda Y, Working Group E. 2024. Complete genome sequence of eight LEE-negative Shiga toxin-producing *Escherichia coli* strains isolated from patients with hemolytic-uremic syndrome. Microbiol Resour Announc 13:e00591-23.

13. Montero DA, Velasco J, Del Canto F, Puente JL, Padola NL, Rasko DA, Farfán M, Salazar JC, Vidal R. 2017. Locus of Adhesion and Autoaggregation (LAA), a pathogenicity island present in emerging Shiga Toxin–producing Escherichia coli strains. Sci Rep 7:7011.

14. Montero DA, Canto FD, Velasco J, Colello R, Padola NL, Salazar JC, Martin CS, Oñate A, Blanco J, Rasko DA, Contreras C, Puente JL, Scheutz F, Franz E, Vidal RM. 2019. Cumulative acquisition of pathogenicity islands has shaped virulence potential and contributed to the emergence of LEE-negative Shiga toxin-producing *Escherichia coli* strains. Emerging Microbes & Infections 8:486–502.

15. Vélez MV, Colello R, Nieto MV, Paz LE, Etcheverría AI, Vidal R, Padola NL. 2024. Transcription levels of hes and their involvement in the biofilm formation of Shiga toxin-producing Escherichia coli O91. Vet Res Commun 48:1821–1830.

16. Ingersoll MA, Moss JE, Weinrauch Y, Fisher PE, Groisman EA, Zychlinsky A. The ShiA protein encoded by the Shigella flexneri SHI-2 pathogenicity island attenuates inflammation.

17. Ingersoll MA, Zychlinsky A. ShiA Abrogates the Innate T-Cell Response to Shigella flexneri Infection https://doi.org/10.1128/iai.74.4.2317-2327.2006.

18. Mao B-H, Chang Y-F, Scaria J, Chang C-C, Chou L-W, Tien N, Wu J-J, Tseng C-C, Wang M-C, Chang C-C, Hsu Y-M, Teng C-H. 2012. Identification of Escherichia coli Genes Associated with Urinary Tract Infections. Journal of Clinical Microbiology https://doi.org/10.1128/jcm.00640-11.

19. Lloyd AL, Smith SN, Eaton KA, Mobley HLT. 2009. Uropathogenic Escherichia coli Suppresses the Host Inflammatory Response via Pathogenicity Island Genes sisA and sisB. Infection and Immunity https://doi.org/10.1128/iai.00779-09.

20. Milani G, Belloso Daza MV, Cortimiglia C, Bassi D, Cocconcelli PS. 2023. Genome engineering of Stx1-and Stx2-converting bacteriophages unveils the virulence of the dairy isolate Escherichia coli O174:H2 strain UC4224. Front Microbiol 14. https://doi.org/10.3389/fmicb.2023.1156375

21. Krause M, Barth H, Schmidt H. 2018. Toxins of Locus of Enterocyte Effacement-Negative Shiga Toxin-Producing Escherichia coli. Toxins 10:241. https://doi.org/10.3390/toxins10060241

22. Tarr CL, Nelson AM, Beutin L, Olsen KEP, Whittam TS. 2008. Molecular Characterization Reveals Similar Virulence Gene Content in Unrelated Clonal Groups of Escherichia coli of Serogroup O174 (OX3). Journal of Bacteriology. https://doi.org/10.1128/jb.01317-07.

23. Steyert SR, Sahl JW, Fraser CM, Teel LD, Scheutz F, Rasko DA. 2012. Comparative Genomics and stx Phage Characterization of LEE-Negative Shiga Toxin-Producing Escherichia coli. Front Cell Inf Microbio 2. https://doi.org/10.3389/fcimb.2012.00133

24. Rodwell EV, Greig DR, Godbole G, Jenkins C. 2024. Clinical and public health implications of increasing notifications of LEE-negative Shiga toxin-producing Escherichia coli in England, 2014–2022. Journal of Medical Microbiology 73. https://doi.org/10.1099/jmm.0.001790

25. Vogeleer P, Tremblay YDN, Jubelin G, Jacques M, Harel J. 2016. Biofilm-Forming Abilities of Shiga Toxin-Producing Escherichia coli Isolates Associated with Human Infections. Appl Environ Microbiol 82:1448–1458. https://doi.org/10.1128/AEM.02983-15

26. Tran M-L, Delannoy S, Fach P. 2025. Enhancing detection of STEC in the meat industry: insights into virulence of priority STEC. Front Microbiol 16. https://doi.org/10.3389/fmicb.2025.1543686

27. Eklund M, Leino K, Siitonen A. 2002. Clinical *Escherichia coli* Strains Carrying *stx* Genes: *stx* Variants and *stx* -Positive Virulence Profiles. J Clin Microbiol 40:4585–4593. https://doi.org/10.1128/jcm.40.12.4585-4593.2002

28. Kawano K, Okada M, Haga T, Maeda K, Goto Y. 2008. Relationship between pathogenicity for humans and stx genotype in Shiga toxin-producing Escherichia coli serotype O157. Eur J Clin Microbiol Infect Dis 27:227–232.

29. Desvaux M, Dalmasso G, Beyrouthy R, Barnich N, Delmas J, Bonnet R. 2020. Pathogenicity Factors of Genomic Islands in Intestinal and Extraintestinal Escherichia coli. Front Microbiol 11:2065.

30. Dutta S, Pazhani GP, Nataro JP, Ramamurthy T. 2015. Heterogenic virulence in a diarrheagenic *Escherichia coli*: Evidence for an EPEC expressing heat-labile toxin of ETEC. International Journal of Medical Microbiology 305:47–54.

31. Bai X, Zhang J, Ambikan A, Jernberg C, Ehricht R, Scheutz F, Xiong Y, Matussek A. 2019. Molecular Characterization and Comparative Genomics of Clinical Hybrid Shiga Toxin-Producing and Enterotoxigenic Escherichia coli (STEC/ETEC) Strains in Sweden. Sci Rep 9:5619.

32. Gati NS, Middendorf-Bauchart B, Bletz S, Dobrindt U, Mellmann A. 2019. Origin and Evolution of Hybrid Shiga Toxin-Producing and Uropathogenic Escherichia coli Strains of Sequence Type 141. J Clin Microbiol 58:e01309-19.

33. Farfan MJ, Torres AG. 2012. Molecular Mechanisms That Mediate Colonization of Shiga Toxin-Producing Escherichia coli Strains. Infect Immun 80:903–913.

34. Govindarajan DK, Viswalingam N, Meganathan Y, Kandaswamy K. 2020. Adherence patterns of Escherichia coli in the intestine and its role in pathogenesis. Medicine in Microecology 5:100025.

35. Colello R, Vélez MV, Farias MVN, Rodríguez M, Montero D, Vidal R, Etcheverría AI, Padola NL. 2023. Expression of hes, iha, and tpsA codified in locus of adhesion and autoaggregation and their involvement in the capability of shiga toxin-producing Escherichia coli strains to adhere to epithelial cells. BMC Res Notes 16:163.

36. Roberfroid S, Vanderleyden J, Steenackers H. 2016. Gene expression variability in clonal populations: Causes and consequences. Critical Reviews in Microbiology 42:969–984.

37. Tsai CJ-Y, Loh JMS, Proft T. 2016. *Galleria mellonella* infection models for the study of bacterial diseases and for antimicrobial drug testing. Virulence 7:214–229.

38. Datta S, Costantino N, Court DL. 2006. A set of recombineering plasmids for gram-negative bacteria. Gene 379:109–115.

39. Serra-Moreno R, Acosta S, Hernalsteens JP, Jofre J, Muniesa M. 2006. Use of the lambda Red recombinase system to produce recombinant prophages carrying antibiotic resistance genes. BMC Molecular Biol 7:31.

40. Mekapogu AR. 2016. Calculating LD50/LC50 using Probit Analysis. https://probitanalysis.wordpress.com/. Retrieved 2 October 2025.

41. Bassi D, Cappa F, Gazzola S, Orrù L, Cocconcelli PS. 2017. Biofilm Formation on Stainless Steel by Streptococcus thermophilus UC8547 in Milk Environments Is Mediated by the Proteinase PrtS. Appl Environ Microbiol 83:e02840-16.

42. Tuomola (Née Lehto) EM, Salminen SJ. 1998. Adhesion of some probiotic and dairy Lactobacillus strains to Caco-2 cell cultures. International Journal of Food Microbiology 41:45–51.

Re: Spectrum03352-25R1 (**Locus of Adhesion and Autoaggregation (LAA) pathogenicity island genes *hes* and *sisA* are involved in virulence and biofilm formation in LEE-negative Shiga-toxin producing *Escherichia coli* (STEC)**)

Dear Dr. Claudia Cortimiglia:

Thank you for supplying the point-by-point responses. Please note the section below describing Data Availability.

Your manuscript has been accepted, and I am forwarding it to the ASM production staff for publication. Your paper will first be checked to make sure all elements meet the technical requirements. ASM staff will contact you if anything needs to be revised before copyediting and production can begin. Otherwise, you will be notified when your proofs are ready to be viewed.

**Data Availability:** ASM policy requires that data be available to the public upon online posting of the article, so please verify all links to sequence records, if present, and make sure that each number retrieves the full record of the data. If a new accession number is not linked or a link is broken, provide production staff with the correct URL for the record. If the accession numbers for new data are not publicly accessible before the expected online posting of the article, publication may be delayed; please contact ASM production staff immediately with the expected release date.

**Publication Fees:** For information on publication fees and which article types have charges, please visit our website. We have partnered with Copyright Clearance Center (CCC) to collect author charges. If fees apply to your paper, you will receive a message from no-reply@copyright.com with further instructions. For questions related to paying charges through RightsLink, please contact CCC at ASM_Support@copyright.com or toll free at +1-877-622-5543. CCC makes every attempt to respond to all emails within 24 hours.

**ASM Membership:** Corresponding authors may join or renew ASM membership to obtain discounts on publication fees. Need to upgrade your membership level? Please contact Customer Service at Service@asmusa.org.

**Embargo Policy:** A press release may be issued as soon as the manuscript is posted on the Spectrum Latest Articles webpage. The corresponding author will receive an email with the subject line "ASM Journals Author Services Notification" when the article is available online.

The ASM Journals program strives for constant improvement in our submission and publication process. Please tell us how we can improve your experience by taking this quick Author Survey.

Thank you for submitting your paper to Spectrum.

Sincerely,
Gillian Tarr
Editor
Microbiology Spectrum
